# Responses of Biocrust and Associated Soil Bacteria to Novel Climates Are Not Tightly Coupled

**DOI:** 10.3389/fmicb.2022.821860

**Published:** 2022-04-28

**Authors:** Anita Antoninka, Peter F. Chuckran, Rebecca L. Mau, Mandy L. Slate, Brent D. Mishler, Melvin J. Oliver, Kirsten K. Coe, Llo R. Stark, Kirsten M. Fisher, Matthew A. Bowker

**Affiliations:** ^1^School of Forestry, Northern Arizona University, Flagstaff, AZ, United States; ^2^Department of Biological Sciences, Center for Ecosystem Science and Society (ECOSS), Northern Arizona University, Flagstaff, AZ, United States; ^3^Department of Ecology and Evolutionary Biology, University of Colorado, Boulder, CO, United States; ^4^Department of Integrative Biology, University and Jepson Herbaria, University of California, Berkeley, Berkeley, CA, United States; ^5^Interdisciplinary Plant Group, Division of Plant Sciences, University of Missouri, Columbia, MO, United States; ^6^Department of Biology, Middlebury College, Middlebury, VT, United States; ^7^School of Life Sciences, University of Nevada, Las Vegas, Las Vegas, NV, United States; ^8^Department of Biological Sciences, California State University, Los Angeles, CA, United States

**Keywords:** common garden, climate change, biological soil crust, community stability, dryland, mosses, lichens, bacterial diversity

## Abstract

Climate change is expanding drylands even as land use practices degrade them. Representing ∼40% of Earth’s terrestrial surface, drylands rely on biological soil crusts (biocrusts) for key ecosystem functions including soil stability, biogeochemical cycling, and water capture. Understanding how biocrusts adapt to climate change is critical to understanding how dryland ecosystems will function with altered climate. We investigated the sensitivity of biocrusts to experimentally imposed novel climates to track changes in productivity and stability under both warming and cooling scenarios. We established three common gardens along an elevational-climate gradient on the Colorado Plateau. Mature biocrusts were collected from each site and reciprocally transplanted intact. Over 20 months we monitored visible species composition and cover, chlorophyll a, and the composition of soil bacterial communities using high throughput sequencing. We hypothesized that biocrusts replanted at their home site would show local preference, and biocrusts transplanted to novel environments would maintain higher cover and stability at elevations higher than their origin, compared to at elevations lower than their origin. We expected responses of the visible biocrust cover and soil bacterial components of the biocrust community to be coupled, with later successional taxa showing higher sensitivity to novel environments. Only high elevation sourced biocrusts maintained higher biocrust cover and community stability at their site of origin. Biocrusts from all sources had higher cover and stability in the high elevation garden. Later successional taxa decreased cover in low elevation gardens, suggesting successional reversal with warming. Visible community composition was influenced by both source and transplant environment. In contrast, soil bacterial community composition was not influenced by transplant environments but retained fidelity to the source. Thus, responses of the visible and soil bacterial components of the biocrust community were not coupled. Synthesis: Our results suggest biocrust communities are sensitive to climate change, and loss of species and function can be expected, while associated soil bacteria may be buffered against rapid change.

## Introduction

Drylands, characterized by limited precipitation and an aridity index of less than 0.65, are among the most important and degraded terrestrial landscapes worldwide (Reynolds et al., 2007; Okin et al., 2011). These ecosystems are important because they encompass more than 40% of the Earth’s terrestrial surface, hold an estimated 25% of Earth’s terrestrial carbon, and support 38% of the world’s population (Reynolds et al., 2007; Prǎvǎlie, 2016). Because drylands are subject to land use activities such as grazing, farming, and mineral extraction, and because of relatively low inherent fertility and productivity, they are particularly susceptible to soil degradation (Austin et al., 2004). Climate change, with increasing temperatures and potential evapotranspiration, or more variable rainfall, further threatens dryland ecosystem health and productivity. Reflective of these challenges, a priority of the United Nations Sustainable Development Goals is to directly address land degradation, including desertification, in drylands (SDG 15, 2015).

Biocrusts are critical to ecosystem function in drylands, often dominating ground cover and filling the interspaces between and under plant canopies (Yeager et al., 2004; Housman et al., 2006). These communities of cyanobacteria, algae, bryophytes and lichens, and other associated soil bacteria and microorganisms living and binding the soil surface, cover approximately 12% of the total global terrestrial surface (Weber et al., 2016; Rodriguez-Caballero et al., 2018). Biocrusts are responsible for many vital ecosystem functions, including primary production, nitrogen (N) fixation, aggregating soil, and regulating hydrologic function (Belnap and Eldridge, 2001; Belnap, 2002; Chamizo et al., 2017; Eldridge et al., 2020).

Biocrusts are tolerant of stress but are susceptible to specific global change stressors. Biocrusts can suspend metabolic activity during periods of no moisture and can rapidly reactivate, responding to small rainfall events (Coe et al., 2012), which makes them able to inhabit soils that are dry most of the time. However, less rainfall or greater evapotranspiration due to higher temperatures can decrease the duration of hydration periods, thereby constricting the time available to biocrusts for metabolic activity and growth. Biocrusts are also susceptible to land uses that physically disturb the soil surface (Ferrenberg et al., 2015). Both physical disturbance and climate change have been shown to reduce the diversity and cover of lichens and mosses, which greatly impacts the ecosystem functions that biocrusts perform (Reed et al., 2012; Ferrenberg et al., 2015; Tucker and Reed, 2016; Ferrenberg et al., 2017; Chuckran et al., 2020). For example, biocrust losses interact with the plant community by modifying albedo, altering soil temperature, creating openings for invasive species, and reducing the net primary productivity and soil fertility *via* loss of activity and through soil loss (Tucker and Reed, 2016; Rutherford et al., 2017; Xiao and Bowker, 2020).

Biocrusts follow predictable patterns of succession after disturbance. Recovery depends upon the nature of the disturbance, soil type and climate (Reed et al., 2012; Weber et al., 2016). On the Colorado Plateau, filamentous cyanobacteria are the first to colonize, stabilizing the soil with their polysaccharide sheaths and making habitat for later successional taxa. Nitrogen-fixing darkly pigmented cyanobacteria come next, followed first by ruderal mosses (e.g., *Bryum* spp.) and lichens (e.g., *Enchylium* spp.), and finally late successional mosses (e.g., *Syntrichia* spp.) and lichens (e.g., *Placydium* spp.; Belnap et al., 2008). In the same way, we see successional retrogression following physical or climate induced disturbance, with loss of taxa from the latest successional groups first (Ferrenberg et al., 2015).

Biocrusts also support a diverse taxonomic and functional soil microbial community, which further expands the list of ecosystem functions facilitated by biocrusts *via* their associated soil microbes (Moreira-Grez et al., 2019), which are tightly associated with the visible biocrust community (Delgado-Baquerizo et al., 2013; Maier et al., 2014). Individual biocrust species support unique soil microbiomes (Moreira-Grez et al., 2019) which impact the functional diversity of the soil microbial community as well (Delgado-Baquerizo et al., 2013). Associations between biocrust and soil microbial communities are also affected by changing climate. For example, Liu et al. (2017) showed that lichen taxa are affected by watering frequency and these changes in the lichen biomass translated to changes in the composition of the associated soil microbial community. Biocrust mosses are also susceptible to warming and altered precipitation (Ferrenberg et al., 2015), but can buffer the soil microbial community composition from aridity (Delgado-Baquerizo et al., 2018). Thus, soil microbial communities associated with biocrusts might respond very little (through buffering) or can change composition, productivity, and function as biocrust communities shift with climate change. These climate-based influences vary depending upon the starting community and the environmental conditions.

While many biocrust taxa are ubiquitous across drylands, the composition varies among and within drylands. We do not know if biocrust taxa, including the microbial community, have the plasticity necessary to adjust to novel climates immediately or if they contain genetic variation sufficient to adapt longer term (Garcia-Pichel et al., 2013; Massatti et al., 2018). Assessing the ability of biocrusts to acclimate to novel climates represents a first step in managing biocrusts in a changing landscape. To determine how biocrust communities respond to novel environments, we set up three common gardens on the Colorado Plateau which is predicted to have higher temperature (4–8^°^C) and lower (∼10%) and more variable precipitation (IPCC., 2014). We reciprocally transplanting mature intact biocrust communities along an elevational and climate gradient reflecting predicted precipitation and temperature changes on the Colorado Plateau (Table 1). We measured “visible biocrust communities” with ocular cover estimates, including lichen, mosses and cyanobacteria to the highest taxonomic level possible without destructive sampling, and “soil bacterial communities,” including the cyanobacterial community, using high throughput sequencing on replicate shallow soil cores. We tested the following hypotheses:

**TABLE 1:** Garden location and site details.

Site	Latitude	Longitude	Elevation (m)	MAP (mm)	MAT (^°^C)	Soil order	Dominant soil series
**Low**	38^°^47’58.50″N	109^°^10’53.53″W	1,291	82	13	Entisol	Ustic Torriorthents
**Mid**	38^°^4’14.31″N	109^°^33’54.61″W	1,627	127	11	Entisol	Redbank
**High**	37^°^59’28.76″’N	109^°^29’6.84″W	2,034	258	10	Entisol	Redbank/Ustic Torriorthents

1. Biocrusts replanted at their home garden will show local preference, with higher cover and greater community stability than in a novel garden.

2. Biocrusts transplanted to a novel garden will maintain higher cover and community stability at elevations higher than their origin, and lower cover and community stability at elevations lower than their origin because of physiological constraints of higher temperatures and lower moisture. Thus, successional reversal of biocrust communities will occur in response to transplantation in warmer environments.

3. Changes in tightly coupled soil bacterial communities will track changes in visible components of biocrust cover and composition.

This information will be valuable to understand how biocrusts adapt to novel environments as well as in a restoration context. We can potentially use assisted migration techniques to move biocrusts to cooler wetter environments in an effort to prepare for future change (i.e., pre-storation; Butterfield et al., 2017).

## Materials and Methods

### Site Selection

In May of 2015 we set up three transplant common gardens arrayed along an elevational-climate gradient, each with fencing protection. We established common gardens in southwestern Utah on the Colorado Plateau at: (1) Bonderman Field Station (low elevation), (2) the Canyonlands Research Center (mid elevation), and (3) a fenced enclosure near the Canyonlands Research Center (high elevation; Table 1). We selected sites to optimize climate differences while limiting soil differences. Sites were all sandy soils with sandstone parent materials (Table 1). We calculated mean annual temperature and precipitation using on-site weather stations (operated by the field stations and the USGS) for 20-month period of the experiment (Table 1).

### Biocrust Collection and Experimental Design

We collected intact, naturally occurring biocrusts at each common garden site, and either replanted them at their site of origin or transported and transplanted them to the other sites. We collected intact biocrusts using 15 cm × 15 cm by 5 cm deep sheet metal samplers. To do this, we carefully pressed samplers into a biocrust moistened with a spray bottle and then applied water until the top several cm of soil were moist as well. The purpose of wetting the soil was to prevent the biocrust and soil from cracking. With a rubber mallet, we inserted the sampler the rest of the way into the soil. We carefully removed the soil around one side to allow the insertion of a bottom tray to hold the biocrust together. We labeled each sample by source garden site and taped the tray to the bottom of the sampler. We selected 45 biocrusts from each garden location that represented each site in terms of a late successional biocrust community (total of 135 transplants; Supplementary Figure 1). We randomly selected 15 samples from each garden location for planting into each of the three gardens, for a total of 45 planted samples per garden including 15 home, and 15 samples from each of the two different elevation sites. We bordered each sample with 6 cm tall vinyl flashing that we carefully folded and bent to slide into the sampler, with 1 cm of flashing exposed aboveground (Supplementary Figure 1). The goal of the flashing was to separate the biocrust from the immediate soil environment and to reduce the potential of being buried *via* overland flow. Transplantation has the artifact of separating from the surrounding soil communities and environment, thus, results should be interpreted in the context of isolated patches.

At each garden, we placed biocrust samples in a random order in three rows of 15 with 50 cm gaps between samples and 1 m gaps between rows. Prior to installation, we cleared vegetation and excavated soil with biocrust samplers to ensure that the surfaces of biocrust transplants would be flush with ground level. We removed the metal samplers and carefully pushed soil to the edges of each biocrust unit and tamped it down to make sure there was good contact with the vinyl flashing and surrounding soil. We stored biocrusts wet and in shade to avoid damaging them until planting, which occurred within 3 days of collection. After planting, we installed light gray polyvinyl weed cloth (Dewitt #CSP350GREY 3 × 50 Gray Weed Fabric) between samples to reduce soil movement and prevent weeds from growing. We completed garden installation in September 2015 and conducted the last sampling in May 2017. Some vascular plants germinated in experimental units, which we carefully clipped above the soil surface at each sampling.

### Measurements

At the time of establishment, and annually thereafter, we collected ocular cover estimates of visible biocrust cover using a gridded frame with each square representing 4% cover. We included mosses, lichens and cyanobacteria, to species level, when possible. We used a hand lens to identify taxa to the highest resolution possible without destructive harvest. Some taxa were identifiable only to the level of genus, or in the case of cyanobacteria, light or dark pigmented community types. Light pigmented cyanobacteria are generally filamentous and live below the soil surface when inactive, whereas dark pigmented cyanobacteria have UV-protective pigments that allow them to live on the surface and appear black when dry, however, both are visible with ocular estimates on the soil surface (Belnap et al., 2008). At the start and end points, we also collected three 1 cm by 0.5 cm depth cores in standardized locations (different for each collection date to avoid previous sampling disturbance) from each experimental unit and homogenized, which were used to estimate chlorophyll *a* content, which is a good proxy for biocrust biomass, and to characterize the soil bacterial community using high throughput sequencing techniques. We air dried samples and stored them frozen until analysis. Soil was homogenized within a biocrust unit and split for chlorophyll *a* and molecular methods. We extracted chlorophyll *a* from ground soil samples with ethanol using the methods of Castle et al. (2011). All measurements were done when biocrusts without wetting to avoid causing unintended stress through short wetting events.

### Molecular Methods

We extracted DNA from all soil samples using a MoBio PowerSoil-htp kit (QIAGEN, United States) following the manufacturer’s instructions. We quantified and assessed the quality of the extracted DNA using a NanoDrop 1,000 spectrophotometer (Thermo Fisher Scientific, United States). Quantitative PCR was performed on each sample to calculate the bacterial 16S rRNA gene copies using primers Eub338F (5’-ACTCCTACGGGAGGCAGCAG-3’) and Eub518R (5’- ATTACCGCGGCTGCTGG-3’) (Fierer and Jackson, 2006). We ran samples in triplicate 10 μL reactions containing 1X Forget-Me-Not EvaGreen qPCR master mix, 1.5 mM MgCl_2_ and 0.25 μM of each primer. We used a Bio-Rad CFX 384 instrument with the following program: 2 min at 95°C, followed by 40 cycles of 95°C for 10 s, 62°C for 10 s, and 72°C for 10 s.

To characterize bacterial community composition, we sent these environmental DNA samples to the Arizona State University Microbiome Analysis Laboratory. We were able to run 10 replicates from all source by garden combination for the initial and end dates. Samples were sequenced using an Illumina platform with primers targeting the V3-V4 hypervariable 16S rRNA region (515F-5’-GTGCCAGCMGCCGCGGTAA—3’ & 806R - 5’-GGACTACHVGGGTWTCTAAT-3’; Caporaso et al., 2012). We imported demultiplexed sequences into QIIME2 (v. 2019.11; Bolyen et al., 2019). We used DADA2 to denoise sequences, join reads, and filter out chimeras (Callahan et al., 2016). We assigned sequence taxonomy using a QIIME2 provided pre-trained Naïve Bayes classifier on the Silva 132 99% OTUs database from the 515F/806R region of sequences using the “feature-classifier classify-sklearn” command in QIIME2 (Quast et al., 2014; Bolyen et al., 2019). We removed all mitochondrial and chloroplast sequences (18% of total sequences) and rarefied samples to 14,595 sequences to standardize sequencing depth and minimize sample loss (Supplementary Figure 2). Raw sequencing reads were uploaded to NCBI’s Sequence Read Archive (SRA) under BioProject number PRJNA806170

### Analysis

To determine if the visible biocrust and soil bacterial communities were different among gardens and sources at the beginning of the study, we used a permutational multivariate analysis of variance (PERMANOVA) for multivariate data and Repeated measures ANOVA for univariate data (H1 and 2). PERMANOVAs were conducted using Bray-Curtis dissimilarity matrices of the square root transformed abundance of each taxon, with 999 permutations. PERMANOVAs were conducted using the *vegan* (Oksanen et al., 2019) package and ANOVAs using the aov function in R v 3.6. (Team RC, 2018). To visualize community differences, we used non-metric multi-dimensional scaling (NMDS) using Bray-Curtis dissimilarity. Community stability was calculated from changes in composition and cover over time as in Tilman (1999) using the community_stability function in the R package *codyn* (Hallett et al., 2016; H1). All figures were created using the ggplot2 package (Wickham, 2016) in R.

We looked for correlations among the biocrust, total soil bacterial community, and just the cyanobacterial community using Mantel tests (PC ORD 6.0; H3). We applied joint plot vectors with a cut-off of *r* = 0.2 on NMDS plots, overlaying soil bacterial abundances on the biocrust community NMDS, and overlaying the biocrust visible taxa cover on the soil bacteria family abundances NMDS plots to look for interactions among the two community types. Axes were rotated to maximize correlation of the most taxa with one axis, and correlation coefficients are reported for taxa with *r* > 0.2. We looked for correlations among visible cyanobacteria cover and the relative abundance of cyanobacteria in the soil samples with simple regression (H3). We also used indicator species analysis to determine if particular taxa were indicative of a particular source, garden or source by garden (PC-ORD 6.0).

To determine directionality of change of each visible biocrust functional group for each source and garden (H2), we calculated the % change in cover from the start and from the home condition as follows:

$$C\,h\,a\,n\,g\,e\,f\,r\,o\,m\,I\,n\,i\,t\,i\,a\,l\,\left(C\,I\right)=\frac{averageendcoverage\left(\%\right)-averagestartcoverage\left(\%\right)}{averagestartcoverage\left(\%\right)}$$

$$P\,e\,r\,c\,e\,n\,t\,C\,h\,a\,n\,g\,e\,f\,r\,o\,m\,H\,o\,m\,e=\frac{\left(C\,I_{a\,w\,a\,y}-C\,I_{h\,o\,m\,e}\right)}{C\,I_{h\,o\,m\,e}}*100$$

These calculations were performed on 1,000 bootstrapped subsamples in order to calculate a mean and standard error of change.

## Results

### Visible Biocrust Community

**FIGURE 1:**
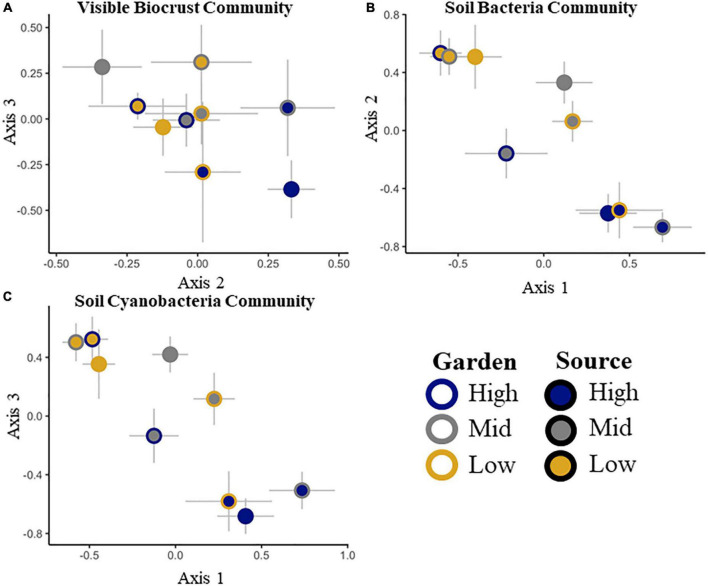
Non-metric multidimensional scaling ordinations of the visible biocrust cover community **(A)**, the total soil bacterial community at the family level **(B)** and the soil cyanobacterial only community at the family level **(C)**. Two of three dimensions are shown, with axes labeled, stress < 0.2 in all cases. Signed axis correlation coefficients are given for taxa correlating at *r* > 0.2, with negative correlations on the left, and positive correlations on the right below each x-axis. No correlations met the criteria for the axis not shown.

To ensure that all gardens received similar biocrust communities from all sources, we tested initial differences among gardens and sources. Initial composition of biocrusts among gardens was not different (pseudo-*F* = 2.1, *p* = 0.2), but all sources had different community compositions (pseudo-*F* = 7.2, *p* = 0.0002). There was no significant source by garden interaction (pseudo-*F* = 0.2, *p* = 0.4; Table 2 and Supplementary Figure 3). After 20 months, we found that the biocrust community composition varied both by source (pseudo-*F* = 8.3, *p* = 0.0002) and by garden (pseudo-*F* = 3.9, *p* = 0.01), with no significant interaction between source and garden (pseudo-*F* = 1.6, *p* = 0.7; Table 2 and Figure 1A). The composition of species overlapped considerably among sources, but the frequency and abundance of taxa varied by source initially and changed through time among sources and gardens (Supplementary Table 1 and Supplementary Figure 3). Among all visible taxa, only dark cyanobacterial cover emerged as an indicator morphotype, indicative of the high elevation source at the initial time point (IV = 25.5, *p* = 0. 003).

**TABLE 2:** Results of PERMANOVA on visible and soil communities at the start and end points.

	Source		Garden		Source X garden	
Start point	Pseudo-*F*	*P*	Pseudo-*F*	*P*	Pseudo-*F*	*P*
Visible cover community	**7.2**	**0.0002**	2.1	0.2	0.1	0.4
Total soil bacterial community (ASV)	**4.2**	**0.001**				
Soil cyanobacterial only community (ASV)	**2.6**	**0.001**				
**End point**						
Visible cover community	**8.3**	**0.0002**	**3.9**	**0.01**	1.6	0.7
Total soil bacterial community (ASV)	**8.6**	**0.001**	0.9	0.7	1.0	0.4
Soil cyanobacterial only community (ASV)	**6.9**	**0.001**	0.7	0.9	1.0	0.44

*Bolded numbers indicate significance at the p ≤ 0.05 level.*

**FIGURE 2:**
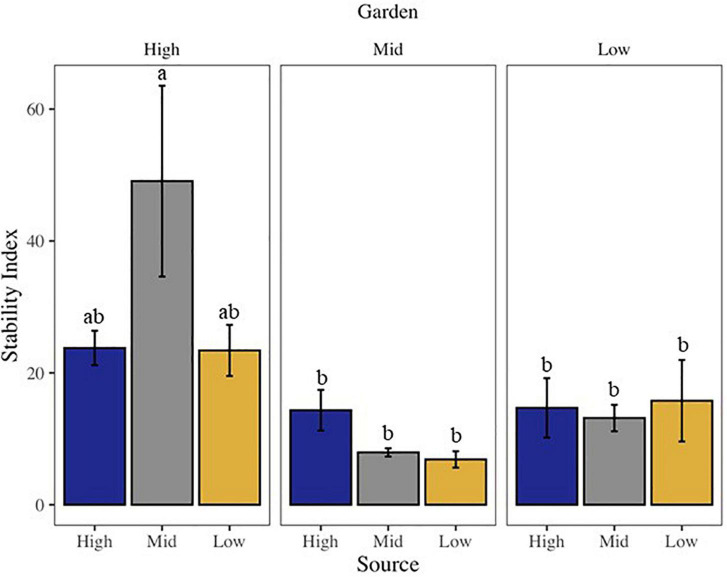
Bar chart of community stability of the visible biocrust community, where higher values indicate greater community stability. Letters above bars indicate differences at *p* < 0.05 with *post-hoc* Tukey’s HSD test.

**FIGURE 3:**
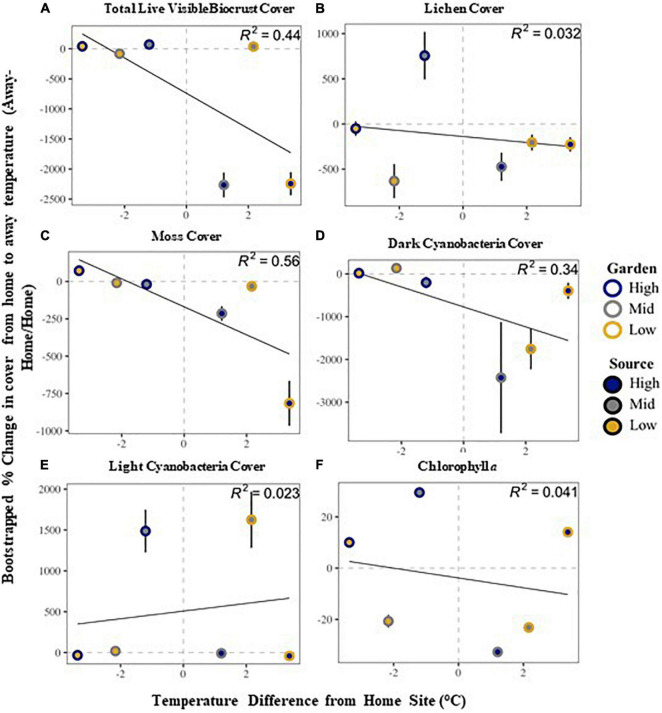
Relationship between visible biocrust cover compositional change from home source to away source based on home-away temperature differences: total live biocrust visible cover **(A)**, lichen cover **(B)**, moss cover **(C)**, dark cyanobacteria cover **(D)**, light cyanobacteria cover **(E)** and chlorophyll a **(F)**. See text in methods for the specific calculations. The solid line is a regression linear fit line representing the difference in temperature from the home site in relationship to the change in cover. The *R*^2^-value is given in the corner of each panel. Dashed line is at zero, and values above that line show an increase, whereas values below demonstrate a decrease. Error bars are bootstrapped standard error.

Community stability was affected by both garden and the interaction of source by garden, with highest stability at the high elevation site among all sources (Supplementary Table 2 and Figure 2). The high elevation site was also the site with the most variability in stability across all sources. All community metrics changed over the 20 months of the experiment, with most metrics decreasing in time, including: overall biocrust visible cover (∼27%), moss cover (∼16%), dark cyanobacteria cover (∼33%), chlorophyll *a* (∼55%), and species richness (∼27%; Supplementary Tables 2, 3). Only bare ground (∼11%) and light cyanobacteria cover increased (∼36%; Supplementary Tables 2, 3). Looking at changes in cover from start to end along the elevation gradient, we found total live cover of all visible biocrust taxa, as well as lichen, moss, and dark cyanobacteria cover, all decreased from high elevation to low elevation over time (Figure 3). However, light cyanobacterial cover and chlorophyll *a* increased from high to low elevation over the course of the experiment (Figure 3 and Supplementary Tables 2, 4).

### Soil Bacterial Community

The initial total soil bacterial and cyanobacterial only communities were different among sources (Table 2 and Supplementary Tables 1, 2). Dominant phyla included Cyanobacteria, Proteobacteria, Actinobacteria, Bacterioidetes, and Acidobacteria (Supplementary Figure 4). We were unable to test for differences in the total bacterial community or the subset of the cyanobacterial community among gardens and sources at the initial point because of damage to some samples, making full comparisons impossible. At the end point, we found differences by source for the whole soil bacterial (pseudo-*F* = 4.6. *p* = 0.001) and cyanobacterial only communities (pseudo-*F* = 5.3, *p* = 0.001), but not by garden or garden by source (Table 2 and Figures 1B,C).

### Associations Among Visible Biocrust and Soil Bacterial Communities

No correlations were found between the visible biocrust community or cyanobacteria visible cover, and the total soil bacterial or soil cyanobacterial only abundances across the sources and gardens (*p* > 0.05). However, there were some associations among communities supported by NMDS axis correlations (Supplementary Table 4). Axis 2 of the biocrust community NMDS (Figure 1A) was correlated with members of the total soil bacterial community, including: unknown Acidobacteria, Coleofasciculaceae (cyanobacteria), Sandaracinaceae (Proteobacteria in the order Myxococcales), Betaproteobacteriales TRA3-20 (Proteobacteria), Acidobacteria, subgroup 6, an uncultured Microtrichales (Actinobacteria), Mycobacteriaceae (Actinobacteria in the order Corynebacteriales), and Verrucomicrobiaceae (Verrucomicrobia, in the order Verrucomicrobiales; Figure 1A and Supplementary Table 4A).

Differences in the total soil bacterial community were correlated with *Syntricia caninervis* along axis 3 (Figure 1B and Supplementary Table 4B). The soil cyanobacterial only community was correlated with light and dark cyanobacterial, along axis 3 (Figure 1C and Supplementary Table 4C). Total number of 16S rRNA gene copies for the total soil bacterial community and the soil cyanobacterial only community both increased with time (Supplementary Tables 2, 3).

## Discussion

### Favorable Conditions Benefit Biocrusts More Than Familiar Environments

Our results suggest that biocrust communities are most amenable to cooler/wetter environments and do not strongly prefer their home environment. This suggests that biocrusts are not necessarily plastic in response to climate but are instead amenable to milder climates than their habitats of origin, supporting hypothesis two, but not hypothesis one. The highest elevation garden had the highest community stability among local and transplanted biocrusts, meaning that biocrusts deviated the least from their starting community composition under cooler, wetter environments. This was especially true for the mid-elevation sourced biocrust and for mosses, lichens, and dark cyanobacteria, compared to the low elevation sourced material, where the decrease in cover was related to increasing temperature and decreasing elevation. Our results are congruent with other experiments eliciting biocrust response to altered temperature and precipitation where successional reversal is induced by warmer climates and more stressful moisture regimes (Escolar et al., 2012; Ferrenberg et al., 2015).

Precipitation appears to be a key driver in biocrust response to novel climates. Higher biocrust cover and stability at the highest elevation garden could be a result of the greater overall precipitation at that site compared to the two lower elevation sites over the course of the experiment. The high elevation garden received more than twice the amount of cool season precipitation of the lower gardens, whereas the mid elevation garden had proportionally higher summer rainfall in comparison to the high elevation garden (73%) and the low elevation garden had only 25% of the total high elevation precipitation. Cool season moisture events are responsible for the majority of carbon fixation and growth in biocrust (Sancho et al., 2016), which suggests that less effective precipitation events occurred in the mid and low elevation gardens. This may account for the lower stability and cover at the mid and low elevation gardens.

Greater stability in the high elevation garden, regardless of source, and the decrease of cover in all home conditions suggests two possible mechanisms: (1) biocrusts are sensitive to transplants and (2) biocrusts may already be maladjusted to their home environments. We expect that transplantation was responsible for some loss, but loss increased with warming and drying. The Colorado Plateau has experienced unprecedented drought and warming over the last three decades (Archer and Predick, 2008), which has led to shifts in plant community composition and some species loss (Munson et al., 2011). Others have documented biocrust loss, particularly for mosses and lichens, in just 1 year of warming and short duration watering (Ferrenberg et al., 2015). Natural dispersal of more stress tolerant phenotypes could plausibly rescue populations of biocrust species, but it is unknown if dispersal rates are adequate to compensate for losses of stress-sensitive phenotypes. Dryland mosses may be increasingly dispersal-limited with climate change due to reduction of sexual reproduction from drought stress and separation of male and female gametophytes (Bowker et al., 2000; Coe et al., 2014). Biocrust lichens must navigate adaptability of both the mycobiont and the photobiont to changing climates. For example, there is evidence from a study with *Psora decipiens* that poor adaptability of the photobiont might limit establishment in novel climates (Williams et al., 2017). As there was heavy overlap in the taxa found among our study sites, we might assume that these taxa are phenotypically plastic; however, it is also possible that locally adapted genotypes are present in different sites. With 30 years of drought and warming, it is possible that biocrust communities on the Colorado Plateau may have already been pushed to the edge of their climatic niche. We also note that transplantation and isolation from the surrounding natural biocrust community limits dispersal and could affect our results.

### Soil Bacterial Communities Did Not Rapidly Shift in Response to Novel Climate

Like the visible biocrust community, we also found the total soil bacterial communities to vary by source. This is not surprising as soil bacterial communities are known to be tightly associated with the biocrust community (Albright et al., 2019), and shaped by environmental conditions (Fierer and Jackson, 2006). However, this community did not shift as expected with transplantation in the same way as the visible biocrust community. Instead, contrary to our prediction in H3, it appears that the total bacterial community response was decoupled from changes in the visible community. These results suggest that the soil community may take longer to respond to novel changes than the visible biocrust community. While unexpected, this is not implausible as the bacteria are mostly below the biocrust or soil surface, and consequently somewhat shielded from the extremes of temperature change, and potentially existing in more hydrologically buffered microclimates compared to the soil surface. There is evidence for this hypothesis in that biocrust mosses buffer microbial communities against soil moisture and aridity-induced changes (Delgado-Baquerizo et al., 2016, 2018).

We expected the cyanobacterial community to be most responsive to changes in the visible biocrust community, but this hypothesis was not well supported. We did see an increase in the abundance of cyanobacterial gene copies across sources and gardens from the start to the end, but it was not strongly correlated with changes in the visible cyanobacteria community. Similarly, the strongest correlation with the total soil bacterial community was with biocrust mosses, and there was little correlation with the visible light and dark cyanobacteria cover. While cyanobacteria associate with biocrust mosses (Adams and Duggan, 2008; Antoninka et al., 2016), they also exist free living and in association with lichens. Perhaps the lack of strong associations result from cyanobacteria occupying a variety of niches. There is some evidence that the biocrust area of greatest influence is at a depth of 0–0.2 cm within the biocrust, whereas we sampled from 0 to 0.5 cm within and below the biocrust (Steven et al., 2013). This is another potential reason for seeing no strong connections between the visible community and the soil cyanobacteria. A third is that the transplantation interfered with local bacterial soil communities and impeding potential shifts that may have occurred. Regardless, our results suggest that the there is some decoupling between soil communities as a function of sampling depth, even when sampling depths differ by only millimeters.

### Implications for Climate Change

Our results are congruent with many others in suggesting that biocrust communities are sensitive to changing climate (Escolar et al., 2012; Ferrenberg et al., 2015), although responses may be decoupled from changes in the soil bacteria community, at least in the short-term. In warming and drying climates, we can expect to lose later successional biocrust cover, and the related functions. Loss of biocrusts in drylands has major implications for ecosystem function and has the potential to lead to further degradation *via* soil erosion and loss of fertility. In this experiment, we saw replacement of mosses and lichens with light cyanobacteria. Perhaps more gradual climate change than experienced with transplantation could ultimately lead to less loss and some adaptation to warming and drying. That this region is in a 30 year drought cycle, with the greatest fluctuations in temperature and precipitation experienced in the past decade. In this context, it is possible that transplanting is within the normal bounds of what they would experience in their home environment. Furthermore, the species overlap among elevations was high, suggesting that natural migration or adaptation might already be occurring. For example, Garcia-Pichel et al. (2013) found that dominant cyanobacterial taxa change with temperature.

The common outcome of successional reversal under climate change or physical disturbance suggests that biocrusts need long term stability to maintain mature and diverse community assemblages and are quickly set back to early successional states, dominated by light pigmented cyanobacteria, by stressful conditions (Reed et al., 2012; Ferrenberg et al., 2015). Loss of dark pigmented cyanobacteria, mosses and lichens can have major implications for ecosystem function. Reduction in photosynthesis and nutrient cycling by biocrusts reduces soil fertility (Lafuente et al., 2018). Increased albedo from the loss of dark pigmented cyanobacteria and lichens influences soil temperature and consequently the growing season for some vascular plans (Rutherford et al., 2017). Loss of biocrust cover decreases soil aggregate stability and can lead to loss of soil and soil fertility (Chiquoine et al., 2016; Antoninka et al., 2017). Biocrusts are critical to function in dryland systems and their loss or successional reversal can have cascading impacts on ecosystem function.

### Implications for Ecological Restoration

Plant ecologists have developed many approaches and policies to mitigate plant extinction due to habitat loss from disturbance or climate change (McLachlan et al., 2007). One controversial approach is assisted migration, where plants or seeds are moved up in elevation or latitude to anticipate the shift of suitable habitat (McLachlan et al., 2007; Gray et al., 2011). Another is “pre-storation,” which advocates using species that will be adapted to both current and future climates in restoration (Butterfield et al., 2017). Assisted migration of biocrusts has been suggested as a possible strategy for biocrust management (Young et al., 2016). Overall, our results suggest that if we wish to restore biocrust materials with a substantial moss or lichen component, we should avoid sourcing the material from a cooler, wetter environment than the restoration site. If we do so, we might expect a loss of these groups or extreme community compositional shifts. Biocrust communities grown in the high elevation environment were the most stable. Thus, if we wish the restored community to closely resemble the inoculum source, the best practice would be to move biocrust inoculum up in elevation. Thus, assisted, or perhaps even natural migration appears to be a viable option to help biocrust communities shift with climate change. Thinking about how to address assisted migration at a meaningful scale and doing so ethically while minimizing biosecurity risks is an important next step.

## Data Availability Statement

The datasets presented in this study can be found in online repositories. The names of the repository/repositories and accession number(s) can be found below: https://data.mendeley.com/datasets/94jg7jkt6n/1; Mendeley Data, https://www.ncbi.nlm.nih.gov/, PRJNA806170.

## Author Contributions

AA implemented the experiment, analyzed the data, and wrote the manuscript. MB and AA conceived the project. PC and RM did molecular analyses. PC made figures. All authors contributed to the ideas and editing.

## Conflict of Interest

The authors declare that the research was conducted in the absence of any commercial or financial relationships that could be construed as a potential conflict of interest.

## Publisher’s Note

All claims expressed in this article are solely those of the authors and do not necessarily represent those of their affiliated organizations, or those of the publisher, the editors and the reviewers. Any product that may be evaluated in this article, or claim that may be made by its manufacturer, is not guaranteed or endorsed by the publisher.

## References

[B1] Adams D. G. Duggan P. S. (2008). Cyanobacteria-bryophyte symbioses. *J. Exp. Bot.* 59 1047–1058. 10.1093/jxb/ern005 18267939

[B2] Albright M. B. N. Mueller R. C. Gallegos-Graves L. V. Belnap J. Reed S. C. Kuske C. R. (2019). Interactions of Microhabitat and Time Control Grassland Bacterial and Fungal Composition. *Front. Ecol. Evol.* 7:367. 10.3389/fevo.2019.00367

[B3] Antoninka A. Bowker M. A. Chuckran P. Barger N. N. Reed S. Belnap J. (2017). Maximizing establishment and survivorship of field-collected and greenhouse-cultivated biocrusts in a semi-cold desert. *Plant Soil* 429 213–225. 10.1007/s11104-017-3300-3

[B4] Antoninka A. Bowker M. A. Reed S. C. Doherty K. (2016). Production of greenhouse-grown biocrust mosses and associated cyanobacteria to rehabilitate dryland soil function. *Restor. Ecol.* 24 3224–3335. 10.1111/rec.12311

[B5] Archer S. A. Predick K. I. (2008). Climate change and ecosystems of the Southwestern United States. *Rangelands* 30 23–38.

[B6] Austin A. T. Yahdjian L. Stark J. M. Belnap J. Porporato A. Norton U. (2004). Water pulses and biogeochemical cycles in arid and semiarid ecosystems. *Oecologia* 141 221–235. 10.1007/s00442-004-1519-1 14986096

[B7] Belnap J. (2002). Impacts of off-road vehicles on nitrogen cycles in biological soil crusts: resistance in different U.S. deserts. *J. Arid Environ.* 52 155–165. 10.1006/jare.2002.0991

[B8] Belnap J. Eldridge D. J. (2001). Disturbance and recovery of biological siol crust. *Ecol. Stud.* 150 363–385. 10.1007/978-3-642-56475-8_27

[B9] Belnap J. Phillips S. L. Witwicki D. L. Miller M. E. (2008). Visually assessing the level of development and soil surface stability of cyanobacterially dominated biological soil crusts. *J. Arid Environ.* 72 1257–1264.

[B10] Bolyen E. Rideout J. R. Dillon M. R. Bokulich N. A. Abnet C. C. Al-Ghalith G. A. (2019). Reproducible, interactive, scalable and extensible microbiome data science using QIIME 2. *Nat. Biotechnol.* 37 852–857. 10.1038/s41587-019-0209-9 31341288 PMC7015180

[B11] Bowker M. A. Stark L. R. McLetchie D. N. Mishler B. D. (2000). Sex expression, skewed sex ratios, and microhabitat distribution in the dioecious desert moss Syntrichia caninervis (Pottiaceae). *Am. J. Bot.* 87 517–526. 10766723

[B12] Butterfield B. J. Copeland S. M. Munson S. M. Roybal C. M. Wood T. E. (2017). Prestoration: using species in restoration that will persist now and into the future. *Restoration Ecology* 25 S155–S163.

[B13] Callahan B. J. McMurdie P. J. Rose M. J. Han A. W. Johnson A. J. A. Holmes S. P. (2016). Dada2: high-resolution sample inference from illumina amplicon data. *Nat. Methods* 13 581–583. 10.1038/nmeth.3869 27214047 PMC4927377

[B14] Caporaso J. G. Lauber C. L. Walters W. A. Berg-Lyons D. Huntley J. Fierer N. (2012). Ultra-high-throughput microbial community analysis on the Illumina HiSeq and MiSeq platforms. *ISME J.* 6 1621–1624. 10.1038/ismej.2012.8 22402401 PMC3400413

[B15] Castle S. C. Morrison C. D. Barger N. N. (2011). Extraction of chlorophyll a from biological soil crusts: a comparison of solvents for spectrophotometric determination. *Soil Biol. Biochem.* 43 853–856. 10.1016/j.soilbio.2010.11.025

[B16] Chamizo S. Rodríguez-Caballero E. Román J. R. Cantón Y. (2017). Effects of biocrust on soil erosion and organic carbon losses under natural rainfall. *Catena* 148 117–125. 10.1016/j.catena.2016.06.017

[B17] Chiquoine L. P. Abella S. R. Bowker M. A. (2016). Rapidly restoring biological soil crusts and ecosystem functions in a severely disturbed desert ecosystem. *Ecol. Appl.* 26 1260–1272. 10.1002/15-0973 27509763

[B18] Chuckran P. F. Reibold R. Throop H. L. Reed S. C. (2020). Multiple mechanisms determine the effect of warming on plant litter decomposition in a dryland. *Soil Biol. Biochem.* 145:107799. 10.1016/j.soilbio.2020.107799

[B19] Coe K. K. Belnap J. Sparks J. P. (2012). Precipitation-driven carbon balance controls survivorship of desert biocrust mosses. *Ecology* 93 1626–1636. 10.1890/11-2247.1 22919909

[B20] Coe K. K. Sparks J. P. Belnap J. (2014). “Physiological Ecology of Dryland Biocrust Mosses,” in *Photosynthesis in Bryophytes and Early Land Plants. Advances in Photosynthesis and Respiration (Including Bioenergy and Related Processes)*, Vol. 37 eds Hanson D. Rice S. (Dordrecht: Springer), 10.1007/978-94-007-6988-5_16

[B21] Delgado-Baquerizo M. Maestre F. T. Eldridge D. J. Bowker M. A. Jeffries T. C. Singh B. K. (2018). Biocrust-forming mosses mitigate the impact of aridity on soil microbial communities in drylands: observational evidence from three continents. *N. Phytol.* 220 824–835. 10.1111/nph.15120 29607501

[B22] Delgado-Baquerizo M. Maestre F. T. Eldridge D. J. Bowker M. A. Ochoa V. Val J. (2016). Biocrust-forming mosses mitigate the negative impacts of increasing aridity on ecosystem multifunctionality in drylands. *N. Phytol.* 209 1540–1552. 10.1111/nph.13688 26452175

[B23] Delgado-Baquerizo M. Morillas L. Maestre F. T. Gallardo A. (2013). Biocrusts control the nitrogen dynamics and microbial functional diversity of semi-arid soils in response to nutrient additions. *Plant Soil* 372 643–654. 10.1007/s11104-013-1779-9

[B24] Eldridge D. J. Reed S. C. Travers S. K. Bowker M. A. Maestre F. T. Ding J. (2020). The pervasive and multifaceted influence of biocrusts on water in the world’s drylands. *Glob. Chang. Biol.* 26 6003–6014. 10.1111/gcb.15232 32729653

[B25] Escolar C. Martinez I. Bowker M. A. Maestre F. T. (2012). Data from: warming reduces the growth and diversity of biological soil crusts in a semi-arid environment: implications for ecosystem structure and functioning. *Phil. Transac. R. Soc. B Biol. Sci.* 367 3087–3099. 10.1098/rstb.2011.0344 23045707 PMC3479686

[B26] Ferrenberg S. Reed S. C. Belnap J. (2015). Climate change and physical disturbance cause similar community shifts in biological soil crusts. *Proc. Natl. Acad. Sci. U.S.A.* 112 12116–12121. 10.1073/pnas.1509150112 26371310 PMC4593113

[B27] Ferrenberg S. Tucker C. L. Reed S. C. (2017). Biological soil crusts: diminutive communities of potential global importance. *Front. Ecol. Environ.* 15:160–167. 10.1002/fee.1469

[B28] Fierer N. Jackson R. B. (2006). The diversity and biogeography of soil bacterial communities. *Nat. Acad. Sci.* 103 626–631. 10.1073/pnas.0507535103 16407148 PMC1334650

[B29] Garcia-Pichel F. Loza V. Marusenko Y. Mateo P. Potrafka R. M. (2013). Temperature Drives the Continental-Scale Distribution of Key Microbes in Topsoil Communities. *Science* 340 1574–1577. 10.1126/science.1236404 23812714

[B30] Gray L. K. Gylander T. Mbogga M. S. Chen P.-Y. Hamann A. (2011). Assisted migration to address climate change: recommendations for aspen reforestation in western Canada. *Ecol. Appl.* 21 1591–1603. 21830704 10.1890/10-1054.1

[B31] Hallett L. M. Jones S. K. MacDonald A. M. Jones M. B. Flynn D. F. B. Rippliner J. (2016). codyn: an R package of community dynamics metrics. *Methods Ecol. Evol.* I, 1146–1151. 10.1111/2041-210X.12569

[B32] Housman D. C. Powers H. H. Collins A. D. Belnap J. (2006). Carbon and nitrogen fixation differ between successional stages of biological soil crusts in the Colorado Plateau and Chihuahuan Desert. *J. Arid Environ.* 66 620–634. 10.1016/j.jaridenv.2005.11.014

[B33] IPCC. (2014). “Climate change 2014: impacts, adaptation, and vulnerability. Part B: regional aspects,” in *Contribution of Working Group II to the Fifth Assessment Report of the Intergovernmental Panel on Climate Change*, eds Barros V. R. Field C. B. Dokken D. J. Mastrandrea M. D. Mach K. J. Bilir T. E. (Cambridge: Cambridge University Press), 688.

[B34] Lafuente A. Berdugo M. Ladrón, de Guevara M. Gozalo B. Maestre F. T. (2018). Simulated climate change affects how biocrusts modulate water gains and desiccation dynamics after rainfall events. *Ecohydrology* 11:e1935. 10.1002/eco.1935 30288205 PMC6166855

[B35] Liu Y. R. Delgado-Baquerizo M. Trivedi P. He J. Z. Wang J. T. Singh B. K. (2017). Identity of biocrust species and microbial communities drive the response of soil multifunctionality to simulated global change. *Soil Biol. Biochem.* 107 208–217. 10.1016/j.soilbio.2016.12.003

[B36] Maier S. Schmidt T. S. B. Zheng L. Peer T. Wagner V. Grube M. (2014). Analyses of dryland biological soil crusts highlight lichens as an important regulator of microbial communities. *Biodivers. Conserv.* 23 1735–1755. 10.1007/s10531-014-0719-1

[B37] Massatti R. Doherty K. D. Wood T. E. (2018). Resolving neutral and deterministic contributions to genomic structure in Syntrichia ruralis (Bryophyta, Pottiaceae) informs propagule sourcing for dryland restoration. *Conserv. Genet.* 19 85–97. 10.1007/s10592-017-1026-7

[B38] McLachlan J. S. Hellmann J. J. Schwartz M. W. (2007). A framework for debate of assisted migration in an era of climate change. *Conserv. Biol.* 21 297–302. 10.1111/j.1523-1739.2007.00676.x 17391179

[B39] Moreira-Grez B. Tam K. Cross A. T. Yong J. W. H. Kumaresan D. Nevill P. (2019). The Bacterial Microbiome Associated With Arid Biocrusts and the Biogeochemical Influence of Biocrusts Upon the Underlying Soil. *Front. Microbiol.* 10:2143. 10.3389/fmicb.2019.02143 31608023 PMC6768011

[B40] Munson S. M. Belnap J. Schelz C. D. Moran M. Carolin T. W. (2011). On the brink of change: plant responses to climate on the Colorado Plateau. *Ecosphere* 2:art68. 10.1890/es11-00059.1

[B41] Okin G. S. Bullard J. E. Reynolds R. L. Ballantine J.-A. C. Schepanski K. Todd M. C. (2011). Dust: small-scale processes with global consequences. *Eos, Transac. Am. Geophys. Union* 92 241–242. 10.1029/2011EO290001

[B42] Oksanen A. J. Blanchet F. G. Kindt R. Legen-P. Minchin P. R. Hara R. B. O. (2019). *vegan: Community Ecology Package.*

[B43] Prǎvǎlie R. (2016). Drylands extent and environmental issues. A global approach. *Ear. Sci. Rev.* 161 259–278. 10.1016/j.earscirev.2016.08.003

[B44] Quast C. Pruesse E. Yilmax P. Gerken J. Schweer T. Yarza P. (2014). The SILVA ribosomal RNA gene database project: improved data processing and web-based tools. *Nucl. Acids Res.* 41 590–596. 10.1093/nar/gks1219 23193283 PMC3531112

[B45] Reed S. C. Coe K. K. Sparks J. P. Housman D. C. Zelikova T. J. Belnap J. (2012). Changes to dryland rainfall result in rapid moss mortality and altered soil fertility. *Nat. Clim. Chang.* 2 752–755. 10.1038/nclimate1596

[B46] Reynolds J. F. Smith D. M. S. Lambin E. F. Turner B. L. Mortimore M. Batterbury S. P. J. (2007). Global Desertification: building a Science for Dryland Development. *Science* 316 847–851. 10.1126/science.1131634 17495163

[B47] Rodriguez-Caballero E. Belnap J. Büdel B. Crutzen P. J. Andreae M. O. Pöschl U. (2018). Dryland photoautotrophic soil surface communities endangered by global change. *Nat. Geosci.* 11 185–189. 10.1038/s41561-018-0072-1

[B48] Rutherford W. A. Painter T. H. Ferrenberg S. Belnap J. Okin G. S. Flagg C. (2017). Albedo feedbacks to future climate via climate change impacts on dryland biocrusts. *Sci. Rep.* 7:44188. 10.1038/srep44188 28281687 PMC5345002

[B49] Sancho L. G. Belnap J. Colesie C. Raggio J. Weber B. (2016). “Budgets of Biological Soil Crusts at Micro-, Meso-, and Global Scales,” in *Biological Soil Crusts: An Organizing Principle in Drylands. Ecological Studies*, Vol. 226 eds Weber B. Büdel B. Belnap J. (Cham: Springer)

[B50] Steven B. Gallegos-Graves L. V. Belnap J. Kuske C. R. (2013). Dryland soil microbial communities display spatial biogeographic patterns associated with soil depth and soil parent material. *FEMS Microbiol. Ecol.* 86 101–113. 10.1111/1574-6941.12143 23621290

[B51] Team RC. (2018). *R: A Language and Environment for Statistical Computing.* Vienna, Austria: R Found Stat Comput.

[B52] Tilman D. (1999). The ecological consequences of changes in biodiversity: a search for general principles. *Ecology* 80 1455–1474. 10.1890/0012-96581999080[1455:TECOCI]2.0.CO;2

[B53] Tucker C. L. Reed S. C. (2016). Low soil moisture during hot periods drives apparent negative temperature sensitivity of soil respiration in a dryland ecosystem: a multi-model comparison. *Biogeochemistry* 128 155–169. 10.1007/s10533-016-0200-1

[B54] Weber B. Bowker M. Zhang Y. Belnap J. (2016). Biological Soil Crusts: an Organizing Principle in Drylands. *Ecol. Stud.* 226 479–498. 10.1007/978-3-319-30214-0

[B55] Wickham H. (2016) *ggplot2: Elegant Graphics for Data Analysisle. 2016.* New York: Springer-Verlag.

[B56] Williams L. Colesie C. Ullmann A. Westberg M. Wedin M. Büdel B. (2017). Lichen acclimation to changing environments: photobiont switching vs. climate-specific uniqueness in Psora decipiens. *Ecol. Evol.* 7 2560–2574. 10.1002/ece3.2809 28428847 PMC5395455

[B57] Xiao B. Bowker M. A. (2020). Moss-biocrusts strongly decrease soil surface albedo, altering land-surface energy balance in a dryland ecosystem. *Sci. Total Environ.* 741:140425. 10.1016/j.scitotenv.2020.140425 32615433

[B58] Yeager C. Kornosky J. Housman D. C. Grote E. E. Belnap J. Kuske C. R. (2004). Diazotrophic community structure and function in two successional stages of biological soil crusts from the Colorado Plateau and Chihuahuan Desert. *Appl. Environ. Microbiol.* 70 973–983. 10.1128/AEM.70.2.973 14766579 PMC348917

[B59] Young K. E. Grover H. S. Bowker M. A. (2016). Altering biocrusts for an altered climate. *N. Phytol.* 210 18–22. 10.1111/nph.13910 26919695

