# Supplementary material for: Responses of Biocrust and Associated Soil Bacteria to Novel Climates Are Not Tightly Coupled
Source: Front Microbiol. 2022 Apr 28;13:821860. doi: 10.3389/fmicb.2022.821860 (PMC9096946; doi:10.3389/fmicb.2022.821860)

**SI**

**Tables**

Table S1. Relative abundance (a) and frequency (b) for the visible cover community at the start (T0) and end point. Light grey indicates the stat, and darker columns indicate the home condition at the end point.

| **a.**  **% Rel abundance** | **Life form** | **Low_T0** | **Low to low** | Low to Mid | Low to High | **Mid_T0** | Mid to Low | **Mid to Mid** | Mid to High | **High_T0** | High to Low | High to Mid | **High to High** |
| --- | --- | --- | --- | --- | --- | --- | --- | --- | --- | --- | --- | --- | --- |
| **Dark Cyanobacteria** | **Cyanobacteria** | **41** | **8** | 9 | 8 | **29** | 9 | **10** | 11 | **30** | 18 | 10 | **17** |
| **Light Cyanobacteria** | **Cyanobacteria** | **59** | **8** | 8 | 7 | **16** | 9 | **6** | 11 | **25** | 11 | 17 | **23** |
| ***Syntrichia ruralis*** | **Moss** | **22** | **16** | 10 | 10 | **39** | 13 | **16** | 6 | **39** | 10 | 10 | **10** |
| ***Syntrichia caninervis*** | **Moss** | **25** | **14** | 5 | 17 | **38** | 19 | **10** | 15 | **37** | 8 | 5 | **6** |
| ***Bryum argenteum*** | **Moss** | **20** | **4** | 24 | 15 | **42** | 5 | **28** | 18 | **39** | 5 | 2 | **0** |
| ***Gemmabryum caespiticium*** | **Moss** | **6** | **16** | 8 | 36 | **54** | 4 | **15** | 17 | **41** | 1 | 2 | **1** |
| ***Bryum kunzei*** | **Moss** | **8** | **0** | 40 | 6 | **36** | 1 | **30** | 16 | **56** | 0 | 6 | **0** |
| ***Encalypta vulgaris*** | **Moss** | **17** | **18** | 21 | 0 | **52** | 7 | **33** | 0 | **31** | 4 | 18 | **0** |
| ***Pterygoneurum sp.*** | **Moss** | **91** | **14** | 9 | 1 | **0** | 15 | **6** | 0 | **9** | 27 | 24 | **4** |
| ***Enchylium tenax*** | **Lichen** | **31** | **16** | 7 | 12 | **33** | 8 | **8** | 19 | **36** | 9 | 7 | **14** |
| ***Enchylium coccophorum*** | **Lichen** | **29** | **19** | 4 | 14 | **33** | 14 | **8** | 24 | **38** | 8 | 4 | **7** |
| ***Candelariella sp.*** | **Lichen** | **66** | **0** | 0 | 6 | **17** | 0 | **8** | 25 | **18** | 3 | 19 | **39** |
| ***Gyalolechia sp.*** | **Lichen** | **44** | **7** | 12 | 4 | **34** | 9 | **4** | 0 | **23** | 7 | 28 | **29** |
| ***Diploschistes muscorum*** | **Lichen** | **73** | **2** | 17 | 26 | **27** | 0 | **4** | 9 | **0** | 7 | 11 | **25** |
| ***Placidium squamulosum*** | **Lichen** | **21** | **8** | 21 | 32 | **60** | 4 | **6** | 5 | **20** | 0 | 19 | **4** |
| ***Psora sp.*** | **Lichen** | **78** | **10** | 0 | 30 | **23** | 0 | **0** | 0 | **0** | 30 | 30 | **0** |
| ***Clavascidium lacinulatum*** | **Lichen** | **12** | **18** | 26 | 18 | **73** | 11 | **6** | 15 | **14** | 0 | 3 | **2** |
| ***Aspicilia sp.*** | **Lichen** | **54** | **0** | 0 | 0 | **29** | 0 | **0** | 100 | **17** | 0 | 0 | **0** |
| ***Endocarpon sp.*** | **Lichen** | **0** | **7** | 0 | 13 | **0** | 0 | **13** | 27 | **100** | 0 | 13 | **27** |
| ***Toninia sp.*** | **Lichen** | **33** | **0** | 31 | 0 | **39** | 63 | **0** | 0 | **29** | 0 | 6 | **0** |
| ***Peltula sp.*** | **Lichen** | **14** | **0** | 0 | 100 | **86** | 0 | **0** | 0 | **0** | 0 | 0 | **0** |
| ***Acarospora sp.*** | **Lichen** | **29** | **0** | 0 | 0 | **57** | 0 | **0** | 0 | **14** | 0 | 100 | **0** |
| **b.**  **% Frequency** |  | **Low_T0** | **Low to low** | Low to Mid | Low to High | **Mid_T0** | Mid to Low | **Mid to Mid** | Mid to High | **High_T0** | High to Low | High to Mid | High to High |
| **Dark Cyanobacteria** | **Cyanobacteria** | **98** | **100** | 93 | 100 | **93** | 80 | **87** | 93 | **96** | 80 | 87 | **100** |
| **Light Cyanobacteria** | **Cyanobacteria** | **96** | **100** | 80 | 100 | **91** | 87 | **80** | 93 | **89** | 93 | 93 | **100** |
| ***Syntrichia ruralis*** | **Moss** | **56** | **80** | 40 | 60 | **93** | 40 | **53** | 27 | **82** | 27 | 40 | **27** |
| ***Syntrichia caninervis*** | **Moss** | **62** | **87** | 60 | 87 | **96** | 80 | **73** | 87 | **93** | 53 | 53 | **47** |
| ***Bryum argenteum*** | **Moss** | **38** | **13** | 67 | 47 | **80** | 40 | **53** | 67 | **87** | 7 | 13 | **0** |
| ***Gemmabryum caespiticium*** | **Moss** | **18** | **40** | 53 | 53 | **62** | 27 | **67** | 67 | **60** | 7 | 13 | **7** |
| ***Gemmabryum kunzei*** | **Moss** | **18** | **0** | 47 | 20 | **62** | 7 | **27** | 40 | **67** | 0 | 13 | **0** |
| ***Encalypta vulgaris*** | **Moss** | **29** | **40** | 33 | 0 | **76** | 13 | **53** | 0 | **69** | 13 | 20 | **0** |
| ***Pterygoneurum sp.*** | **Moss** | **31** | **13** | 13 | 7 | **0** | 20 | **20** | 0 | **4** | 40 | 13 | **7** |
| ***Enchylium tenax*** | **Lichen** | **82** | **80** | 60 | 80 | **87** | 60 | **73** | 80 | **91** | 60 | 80 | **87** |
| ***Enchylium coccophorum*** | **Lichen** | **62** | **40** | 20 | 47 | **64** | 20 | **33** | 60 | **69** | 33 | 27 | **33** |
| ***Candelariella sp.*** | **Lichen** | **33** | **0** | 0 | 7 | **11** | 0 | **13** | 13 | **11** | 7 | 27 | **20** |
| ***Gyalolechia sp.*** | **Lichen** | **22** | **13** | 13 | 7 | **11** | 7 | **7** | 0 | **11** | 13 | 40 | **40** |
| ***Diploschistes muscorum*** | **Lichen** | **9** | **7** | 33 | 33 | **2** | 0 | **7** | 13 | **0** | 13 | 20 | **27** |
| ***Placidium squamulosum*** | **Lichen** | **20** | **13** | 27 | 33 | **40** | 7 | **20** | 13 | **27** | 0 | 27 | **7** |
| ***Psora sp.*** | **Lichen** | **11** | **7** | 0 | 13 | **4** | 0 | **0** | 0 | **0** | 7 | 7 | **0** |
| ***Clavascidium lacinulatum*** | **Lichen** | **7** | **13** | 7 | 33 | **22** | 13 | **13** | 27 | **7** | 0 | 7 | **7** |
| ***Aspicilia sp.*** | **Lichen** | **27** | **0** | 0 | 0 | **11** | 0 | **0** | 7 | **7** | 0 | 0 | **0** |
| ***Endocarpon sp.*** | **Lichen** | **0** | **7** | 0 | 7 | **0** | 0 | **7** | 13 | **4** | 0 | 7 | **13** |
| ***Toninia sp.*** | **Lichen** | **16** | **0** | 7 | 0 | **16** | 13 | **0** | 0 | **9** | 0 | 7 | **0** |
| ***Peltula sp.*** | **Lichen** | **4** | **0** | 0 | 7 | **18** | 0 | **0** | 0 | **0** | 0 | 0 | **0** |
| ***Acarospora sp.*** | **Lichen** | **4** | **0** | 0 | 0 | **2** | 0 | **0** | 0 | **2** | 0 | 7 | **0** |

Table S2. Results of repeated measures ANOVA and ANOVA for all univariate data.

|  | | **Time** | | **Source** | | | **Garden** | | | **Source x Garden** | | | **Source x Time** | | | **Garden x Time** | | | **Source x Garden x Time** | | |
| --- | --- | --- | --- | --- | --- | --- | --- | --- | --- | --- | --- | --- | --- | --- | --- | --- | --- | --- | --- | --- | --- |
|  | **F** | | **P** | | **F** | **P** | | **F** | **P** | | **F** | **P** | | **F** | **P** | | **F** | **P** | | **F** | **P** |
| **Total visible cover** | **89.2** | | **0.0001** | | 0.8 | 0.4 | | **8.9** | **0.002** | | 0.7 | 0.6 | | **2.5** | **0.03** | | **20.7** | **0.0001** | | **2.0** | **0.02** |
| **Dark cyanobacteria cover** | **11.6** | | **0.0001** | | **13.8** | **0.0001** | | 1.0 | 0.4 | | 0.3 | 0.9 | | **9.1** | **0.0001** | | **4.8** | **0.0001** | | 1.4 | 0.2 |
| **Lichen cover** | **13.9** | | **0.0001** | | 0.4 | 0.6 | | **3.3** | **0.04** | | 0.3 | 0.9 | | 1.4 | 0.2 | | **3.5** | **0.003** | | 1.6 | 0.09 |
| **Moss cover** | **20.9** | | **0.0001** | | **17.3** | **0.0001** | | 1.0 | 0.4 | | 1.0 | 0.4 | | 1.7 | 0.1 | | **2.6** | **0.02** | | 0.9 | 0.5 |
| **LC cover** | **32.7** | | **0.0001** | | **32.2** | **0.0001** | | **3.8** | **0.03** | | **3.7** | **0.007** | | **5.3** | **0.0001** | | **4.9** | **0.0001** | | 1.4 | 0.2 |
| **Bare** | **47.0** | | **0.0001** | | **3.5** | **0.03** | | **19.5** | **0.001** | | 1.4 | 0.2 | | 0.8 | 0.6 | | **13.4** | **0.0001** | | 1.3 | 0.2 |
| **chl a** | **45.0** | | **0.0001** | | **4.3** | **0.02** | | **5.0** | **0.008** | | 0.3 | 0.9 | | **4.7** | **0.01** | | 0.0 | 1 | | 0.0 | 1 |
| **species richness (cover)** | **59.0** | | **0.0001** | | **14.0** | **0.0001** | | **9.6** | **0.001** | | 0.6 | 0.7 | | 0.2 | 1 | | **3.0** | **0.007** | | 1.6 | 0.1 |
| **evenness (cover)** | **20.2** | | **0.0001** | | **4.5** | **0.01** | | 1.7 | 0.2 | | 1.0 | 0.4 | | 1.3 | 0.3 | | **2.0** | **0.07** | | 0.8 | 0.7 |
| **QPCR gene copy (ul)* no Mid elevation** | **9.0** | | **0.004** | | 1.7 | 0.2 | | 0.0 | 0.9 | | 2.5 | 0.1 | | 0.4 | 0.7 | | 0.9 | 0.4 | | 0.3 | 0.7 |
| **QPCR cyanobacteria (ul)* no Mid elevation** | **7.0** | | **0.01** | | 0.0 | 1 | | 0.5 | 0.5 | | 0.6 | 0.4 | | 0.2 | 0.7 | | 0.6 | 0.4 | | 0.2 | 0.7 |
| **Stability** |  | |  | | 1.5 | 0.2 | | **12.3** | **<0.0001** | | **2.7** | **0.03** | |  |  | |  |  | |  |  |

Table S3. Means and standard errors for all univariate response variables. Letters after mean (SE) indicate differences with Tukey’s Post-Hoc tests.

| **Source** | **Garden** | **% Live Biocrust Cover_0** | **% Live Biocrust Cover_20** | **% Light Cyanobacteria_0** | **% Light Cyanobacteria_20** | **%Dark Cyanobacteria_0** | **%Dark Cyanobacteria_20** | **% Lichen_0** | **% Lichen_20** | **%Moss_0** | **%Moss_20** | **%Bare_0** | **%Bare_20** |
| --- | --- | --- | --- | --- | --- | --- | --- | --- | --- | --- | --- | --- | --- |
| **Low** | **Low** | 112.9 (2.7) a | 81.0 (5.8) abc | 5.9 (0.7) c | 15.7 (2.1) c | 18.9 (4.8) a | 10.3 (1.4) a | 11.8 (2.4) a | 17.9 (4.5) a | 64.1 (6.4) a | 45.5 (6.3) ab | 0.0 (0.0) a | 1.0 (1.0) a |
| **Low** | **Mid** | 109.1 (4.0) ab | 52.3 (9.1) d | 7.1 (1.6) c | 14.9 (3.6) c | 12.8 (2.7) a | 14.9 (3.9) a | 24.4 (4.7) a | 14.2 (4.0) a | 56.0 (5.9) a | 36.2 (8.2) ab | 0.0 (0.0) a | 2.9 (1.2) a |
| **Low** | **High** | 106.3 (2.4) ab | 87.9 (4.1) ab | 7.1 (1.3) c | 14.4 (2.4) c | 14.4 (2.3) a | 9.3 (1.5) a | 20.6 (4.6) a | 19.8 (4.5) a | 57.6 (6.6) a | 52.5 (5.6) a | 0.7 (0.7) a | 1.1 (0.8) a |
| **Mid** | **Low** | 107.9 (2.5) ab | 82.6 (3.3) abc | 8.1 (2.0) c | 17.2 (3.1) bc | 19.8 (3.6) a | 11.6 (2.4) a | 12.1 (2.8) a | 11.4 (3.6) a | 59.9 (6.3) a | 49.6 (5.8) a | 0.5 (0.5) a | 0.8 (0.6) a |
| **Mid** | **Mid** | 106.1 (1.4) ab | 64.9 (5.0) cd | 10.5 (3.3) bc | 11.1 (3.2) c | 13.7 (3.9) a | 12.9 (3.4) a | 12.0 (2.6) a | 10.5 (2.6) a | 63.8 (5.9) a | 53.3 (6.0) a | 0.5 (0.5) a | 3.6 (1.0) a |
| **Mid** | **High** | 107.7 (2.0) ab | 96.5 (2.0) a | 11.7 (3.0) bc | 20.5 (3.8) bc | 15.5 (2.6) a | 13.8 (2.5) a | 20.1 (2.9) a | 22.0 (4.3) a | 53.0 (6.0) ab | 43.2 (6.9) ab | 0.5 (0.5) a | 0.3 (0.3) a |
| **High** | **Low** | 102.4 (1.0) b | 68.5 (9.7) bcd | 14.9 (3.3) bc | 21.6 (5.1) bc | 25.5 (5.3) a | 22.3 (5.3) a | 12.9 (4.2) a | 9.4 (3.2) a | 45.6 (9.4) ab | 25.2 (7.1) ab | 1.9 (1.7) a | 5.4 (3.6) a |
| **High** | **Mid** | 103.5 (1.5) ab | 70.3 (5.0) bcd | 23.7 (6.0) ab | 33.6 (5.5) ab | 22.7 (4.3) a | 12.3 (2.7) a | 20.2 (3.9) a | 14.0 (3.0) a | 33.5 (9.5) ab | 26.1 (8.3) ab | 0.1 (0.1) a | 4.8 (1.3) a |
| **High** | **High** | 102.7 (1.5) b | 97.0 (3.5) a | 33.5 (4.3) a | 45.2 (4.5) a | 23.1 (3.0) a | 19.8 (2.7) a | 20.4 (4.4) a | 16.7 (3.8) a | 23.6 (7.4) b | 18.0 (6.4) b | 0.7 (0.7) a | 0.0 (0.0) a |
| **Source only** | |  |  |  |  |  |  |  |  |  |  |  |  |
| **Low** | | 109.4 (3.0) a | 73.7 (6.3) a | 6.7 (1.2) b | 15.0 (2.7) b | 15.4 (3.3) b | 11.5 (2.3) b | 18.9 (3.9) a | 17.3 (4.4) a | 59.2 (6.3) a | 44.7 (6.7) a | 0.2 (0.2) a | 1.6 (1.0) a |
| **Mid** | | 107.2 (1.9) a | 81.4 (3.4) a | 10.1 (2.8) b | 16.3 (3.4) b | 16.3 (3.4) b | 12.8 (2.8) ab | 14.7 (2.8) a | 14.6 (3.5) a | 58.9 (6.1) a | 48.7 (6.2) a | 0.5 (0.5) a | 1.6 (0.6) a |
| **High** | | 102.8 (1.3) a | 78.6 (6.1) a | 24.0 (4.5) a | 33.5 (5.0) a | 23.8 (4.2) a | 18.1 (3.6) a | 17.8 (4.2) a | 13.4 (3.3) a | 34.2 (8.7) b | 23.1 (7.3) b | 0.9 (0.8) a | 3.4 (1.6) a |
| **Garden only** | |  |  |  |  |  |  |  |  |  |  |  |  |
| **Low** | | 107.8 (2.0) a | 77.4 (6.3) b | 9.6 (2.0) b | 18.2 (3.4) b | 21.4 (4.6) a | 14.7 (3.1) a | 12.3 (3.1) b | 12.9 (3.8) a | 56.5 (7.3) a | 40.1 (6.4) a | 0.8 (0.7) a | 2.4 (1.7) ab |
| **Mid** | | 105.3 (1.8) a | 74.4 (4.7) c | 13.8 (3.6) ab | 19.7 (3.7) ab | 16.9 (3.5) a | 11.5 (2.5) a | 17.6 (3.7) ab | 14.8 (3.4) a | 51.6 (7.3) a | 44.0 (6.6) a | 0.4 (0.4) a | 3.2 (1.0) a |
| **High** | | 105.5 (1.9) b | 93.8 (3.2) a | 17.4 (2.9) b | 26.7 (3.5) a | 17.6 (2.6) a | 14.3 (2.2) a | 20.4 (4.0) a | 19.5 (4.2) a | 44.7 (6.6) a | 37.9 (6.3) a | 0.6 (0.6) a | 0.4 (0.3) b |

| **Source** | **Garden** |  | **Chlorophyll a (ug/g soil)_0** | **Chlorophyll a (ug/g soil)_20** | **Richness_0** | **Richness_20** | **Evenness_0** | **Evenness_20** | **Shannon's Diversity_0** | **Shannon's Diversity_20** | **Community stability_20** | **Soil Bacteria _0** | **Soil Bacteria _20** | **Soil Cyanobacteria _0** | **Soil Cyanobacteria _20** |  |
| --- | --- | --- | --- | --- | --- | --- | --- | --- | --- | --- | --- | --- | --- | --- | --- | --- |
| **Low** | **Low** |  | 71.1 (5.8) ab | 31.3 (3.3) a | 8.2 (0.6) ab | 6.5 (0.5) ab | 0.7 (0.0) a | 0.8 (0.0) a | 0.7 (0.1) a | 0.7 (0.0) a | 15.8 (6.2) b | 29736921 (4037555) ab | 39091574 (4406358) b | 7089588 (2393532) a | 11932385 (2822792) a |  |
| **Low** | **Mid** |  | 79.2 (8.5) a | 49.5 (4.1) a | 10.5 (0.5) a | 6.5 (0.6) ab | 0.8 (0.0) a | 0.8 (0.0) a | 0.8 (0.0) a | 0.7 (0.0) a | 6.9 (1.2) b | NA | 36227803 (4319450) ab | NA | 11361233 (2051063) a |  |
| **Low** | **High** |  | 97.2 (10.7) ab | 43.4 (6.1) a | 9.5 (0.5) ab | 7.4 (0.5) a | 0.8 (0.0) a | 0.8 (0.0) a | 0.8 (0.0) a | 0.7 (0.0) a | 23.4 (3.9) ab | 21971651 (2336659) ab | 32236099 (5493793) ab | 5350656 (1127451) a | 9270602 (1858115) a |  |
| **Mid** | **Low** |  | 70.8 (4.4) b | 23.0 (1.8) a | 8.1 (0.7) ab | 5.1 (0.5) ab | 0.7 (0.0) a | 0.7 (0.0) a | 0.6 (0.0) a | 0.6 (0.0) a | 13.2 (2.0) b | 22449597 (2580908) ab | 31330082 (4430144) ab | 5125723 (1225125) a | 7238353 (2015437) a |  |
| **Mid** | **Mid** |  | 104.9 (12.5) ab | 42.1 (4.0) a | 8.9 (0.5) ab | 6.9 (0.7) ab | 0.7 (0.0) a | 0.8 (0.1) a | 0.7 (0.0) a | 0.7 (0.1) a | 7.9 (0.6) b | NA | 25785697 (3066006) ab | NA | 6029827 (2081547) a |  |
| **Mid** | **High** |  | 79.6 (9.3) ab | 44.7 (3.7) a | 9.6 (0.5) ab | 7.0 (0.6) a | 0.8 (0.0) a | 0.8 (0.0) a | 0.8 (0.0) a | 0.7 (0.0) a | 49.1 (14.5) a | 30234496 (3364028) ab | 32204302 (5946592) ab | 7692376 (1994624) a | 11438021 (3568452) a |  |
| **High** | **Low** |  | 83.3 (11.9) ab | 35.5 (11.8) a | 7.0 (0.7) b | 4.5 (0.5) b | 0.7 (0.0) a | 0.7 (0.1) a | 0.6 (0.1) a | 0.5 (0.1) a | 23.4 (3.9) ab | 20098974 (3135919) a | 29408305 (3809024) ab | 5726387 (1417783) a | 10756116 (2575949) a |  |
| **High** | **Mid** |  | 87.3 (14.5) ab | 42.8 (5.2) a | 7.6 (0.8) b | 6.0 (0.5) ab | 0.7 (0.0) a | 0.7 (0.0) a | 0.6 (0.1) a | 0.6 (0.0) a | 14.3 (3.1) b | NA | 26954743 (3117659) ab | NA | 7986973 (2189398) a |  |
| **High** | **High** |  | 84.6 (9.4) ab | 39.8 (3.4) a | 7.7 (0.5) b | 5.2 (0.2) ab | 0.8 (0.0) a | 0.8 (0.0) a | 0.7 (0.0) a | 0.6 (0.0) a | 23.8 (2.6) a | 25680988 (2602723) ab | 27820942 (3627255) ab | 7664123 (1960168) a | 9197418 (1893601) a |  |
| **Source only** | |  |  |  |  |  |  |  |  |  |  |  |  |  |  |  |
| **Low** | |  | 82.5 (8.4) a | 41.4 (4.5) a | 9.4 (0.5) a | 6.8 (0.5) a | 0.8 (0.0) a | 0.8 (0.0) a | 0.8 (0.0) a | 0.7 (0.0) a | 15.3 (2.6) a | 25854286 (2438756) ab | 25854286 (2438756)  ab | 35851825 (2708033) b | 6168977 (1250225) a |  |
| **Mid** | |  | 85.1 (8.7) a | 36.6 (3.2) a | 8.8 (0.5) a | 6.3 (0.6) a | 0.7 (0.0) a | 0.8 (0.0) a | 0.7 (0.0) ab | 0.7 (0.0) ab | 23.4 (5.5) a | 26342047 (2248405) ab | 26342047 (2248405) ab | 29773360 (2634281) ab | 6476593 (1205906) a |  |
| **High** | |  | 85.1 (11.9) a | 39.4 (6.8) a | 7.4 (0.7) b | 5.2 (0.4) b | 0.7 (0.0) a | 0.7 (0.0) a | 0.7 (0.0) b | 0.6 (0.0) b | 17.6 (2.1) a | 22889981 (2084105) a | 22889981 (2084105) a | 28061330 (1975615) ab | 6746248 (1219142) a |  |
| **Garden only** | |  |  |  |  |  |  |  |  |  |  |  |  |  |  |  |
| **Low** | |  | 75.1 (7.4) b | 29.9 (5.6) a | 7.8 (0.6) b | 5.4 (0.5) b | 0.7 (0.0) b | 0.8 (0.0) a | 0.7 (0.1) b | 0.6 (0.0) a | 14.5 (2.6) b | 24095164 (1993320) a | 24095164 (1993320) a | 33276654 (2478700) a | 5937912 (953188.2) a |  |
| **Mid** | |  | 96.4 (12.6) a | 42.8 (5.1) a | 8.7 (0.6) a | 6.8 (0.6) a | 0.7 (0.0) ab | 0.8 (0.0) ab | 0.7 (0.0) ab | 0.7 (0.0) a | 9.7 (1.2) b | NA | NA | 29656081 (2158824) a | NA |  |
| **High** | |  | 87.1 (9.8) a | 42.6 (4.4) a | 8.9 (0.5) a | 6.5 (0.4) a | 0.8 (0.0) a | 0.8 (0.0) b | 0.8 (0.0) a | 0.7 (0.0) a | 32.0 (5.3) a | 25962378 (1682272) a | 25962378 (1682272) a | 30753781 (2879194) a | 6955893 (1010428) a |  |

Table S4. Correlation with NMDS axes for the visible biocrust community (a), total soil bacterial community (b) and the soil cyanobacterial only community (c). These values correspond with Figure 1a-c.

| **a.**  **Bacteria best match – correlations with biocrust cover NMDS (Fig. 1a)** | **Axis 1** | **Axis 2 (x)** | **Axis 3 (y)** | |
| --- | --- | --- | --- | --- |
|  | **r-sq** | **r-sq** | **r-sq** | |
|  |  |  |  | |
| D_0__Archaea;D_1__Thaumarchaeota;D_2__Nitrososphaeria;D_3__Nitrososphaerales;D_4__Nitrososphaeraceae | 0.001 | 0.004 | 0.001 | |
| D_0__Bacteria;D_1__Acidobacteria;D_2__Acidobacteriia;D_3__Solibacterales;D_4__Solibacteraceae (Subgroup 3) | 0.014 | 0.148 | 0.039 | |
| D_0__Bacteria;D_1__Acidobacteria;D_2__Blastocatellia (Subgroup 4);D_3__11-24;D_4__uncultured bacterium | 0.013 | 0.086 | 0.015 | |
| D_0__Bacteria;D_1__Acidobacteria;D_2__Blastocatellia (Subgroup 4);D_3__Blastocatellales;D_4__Blastocatellaceae | 0.004 | 0.001 | 0 | |
| D_0__Bacteria;D_1__Acidobacteria;D_2__Blastocatellia (Subgroup 4);D_3__DS-100;__ | 0.001 | 0.005 | 0.005 | |
| D_0__Bacteria;D_1__Acidobacteria;D_2__Blastocatellia (Subgroup 4);D_3__Pyrinomonadales;D_4__Pyrinomonadaceae | 0.004 | 0.104 | 0.018 | |
| D_0__Bacteria;D_1__Acidobacteria;D_2__Holophagae;D_3__Subgroup 7;D_4__uncultured Acidobacteria bacterium | 0.025 | 0.008 | 0.008 | |
| D_0__Bacteria;D_1__Acidobacteria;D_2__Holophagae;D_3__Subgroup 7;__ | 0.002 | 0.019 | 0 | |
| D_0__Bacteria;D_1__Acidobacteria;D_2__Subgroup 6;__;__ | 0.038 | **0.297** | 0.004 | |
| D_0__Bacteria;D_1__Acidobacteria;D_2__Thermoanaerobaculia;D_3__Thermoanaerobaculales;D_4__Thermoanaerobaculaceae | 0.028 | 0.002 | 0.038 | |
| D_0__Bacteria;D_1__Actinobacteria;D_2__0319-7L14;D_3__uncultured bacterium;D_4__uncultured bacterium | 0 | 0.01 | 0.004 | |
| D_0__Bacteria;D_1__Actinobacteria;D_2__Acidimicrobiia;D_3__IMCC26256;__ | 0.028 | 0.001 | 0.005 | |
| D_0__Bacteria;D_1__Actinobacteria;D_2__Acidimicrobiia;D_3__Microtrichales;D_4__Iamiaceae | 0 | 0.001 | 0.005 | |
| D_0__Bacteria;D_1__Actinobacteria;D_2__Acidimicrobiia;D_3__Microtrichales;D_4__Ilumatobacteraceae | 0 | 0.082 | 0.001 | |
| D_0__Bacteria;D_1__Actinobacteria;D_2__Acidimicrobiia;D_3__Microtrichales;D_4__uncultured | 0.006 | **0.231** | 0.008 | |
| D_0__Bacteria;D_1__Actinobacteria;D_2__Acidimicrobiia;D_3__Microtrichales;__ | 0.022 | 0.081 | 0.01 | |
| D_0__Bacteria;D_1__Actinobacteria;D_2__Acidimicrobiia;D_3__uncultured;D_4__uncultured Iamia sp. | 0 | 0.005 | 0.001 | |
| D_0__Bacteria;D_1__Actinobacteria;D_2__Acidimicrobiia;D_3__uncultured;D_4__uncultured bacterium | 0.005 | 0.019 | 0.018 | |
| D_0__Bacteria;D_1__Actinobacteria;D_2__Acidimicrobiia;D_3__uncultured;__ | 0.01 | 0.018 | 0 | |
| D_0__Bacteria;D_1__Actinobacteria;D_2__Actinobacteria;D_3__Corynebacteriales;D_4__Mycobacteriaceae | 0.006 | **0.202** | 0.001 | |
| D_0__Bacteria;D_1__Actinobacteria;D_2__Actinobacteria;D_3__Frankiales;D_4__Cryptosporangiaceae | 0.027 | 0.179 | 0 | |
| D_0__Bacteria;D_1__Actinobacteria;D_2__Actinobacteria;D_3__Frankiales;D_4__Frankiaceae | 0.08 | 0.012 | 0.002 | |
| D_0__Bacteria;D_1__Actinobacteria;D_2__Actinobacteria;D_3__Frankiales;D_4__Geodermatophilaceae | 0.005 | 0.007 | 0.006 | |
| D_0__Bacteria;D_1__Actinobacteria;D_2__Actinobacteria;D_3__Frankiales;D_4__Nakamurellaceae | 0.008 | 0.001 | 0.001 | |
| D_0__Bacteria;D_1__Actinobacteria;D_2__Actinobacteria;D_3__Frankiales;D_4__Sporichthyaceae | 0.033 | 0.005 | 0.025 | |
| D_0__Bacteria;D_1__Actinobacteria;D_2__Actinobacteria;D_3__Frankiales;D_4__uncultured | 0.073 | 0.005 | 0.03 | |
| D_0__Bacteria;D_1__Actinobacteria;D_2__Actinobacteria;D_3__Frankiales;__ | 0.026 | 0.026 | 0.026 | |
| D_0__Bacteria;D_1__Actinobacteria;D_2__Actinobacteria;D_3__Kineosporiales;D_4__Kineosporiaceae | 0.001 | 0.02 | 0 | |
| D_0__Bacteria;D_1__Actinobacteria;D_2__Actinobacteria;D_3__Micrococcales;D_4__Cellulomonadaceae | 0.003 | 0.038 | 0.084 | |
| D_0__Bacteria;D_1__Actinobacteria;D_2__Actinobacteria;D_3__Micrococcales;D_4__Intrasporangiaceae | 0 | 0.002 | 0.004 | |
| D_0__Bacteria;D_1__Actinobacteria;D_2__Actinobacteria;D_3__Micrococcales;D_4__Microbacteriaceae | 0.02 | 0 | 0.002 | |
| D_0__Bacteria;D_1__Actinobacteria;D_2__Actinobacteria;D_3__Micrococcales;D_4__Micrococcaceae | 0 | 0.004 | 0.003 | |
| D_0__Bacteria;D_1__Actinobacteria;D_2__Actinobacteria;D_3__Micromonosporales;D_4__Micromonosporaceae | 0.005 | 0.104 | 0.011 | |
| D_0__Bacteria;D_1__Actinobacteria;D_2__Actinobacteria;D_3__Propionibacteriales;D_4__Nocardioidaceae | 0.008 | 0.028 | 0.001 | |
| D_0__Bacteria;D_1__Actinobacteria;D_2__Actinobacteria;D_3__Pseudonocardiales;D_4__Pseudonocardiaceae | 0 | 0.006 | 0.008 | |
| D_0__Bacteria;D_1__Actinobacteria;D_2__Actinobacteria;D_3__Streptomycetales;D_4__Streptomycetaceae | 0.005 | 0.017 | 0.017 | |
| D_0__Bacteria;D_1__Actinobacteria;D_2__Actinobacteria;D_3__Streptosporangiales;D_4__Streptosporangiaceae | 0.028 | 0.002 | 0 | |
| D_0__Bacteria;D_1__Actinobacteria;D_2__Actinobacteria;__;__ | 0.001 | 0.008 | 0.006 | |
| D_0__Bacteria;D_1__Actinobacteria;D_2__Nitriliruptoria;D_3__Euzebyales;D_4__Euzebyaceae | 0.009 | 0.003 | 0 | |
| D_0__Bacteria;D_1__Actinobacteria;D_2__Rubrobacteria;D_3__Rubrobacterales;D_4__Rubrobacteriaceae | 0 | 0.014 | 0 | |
| D_0__Bacteria;D_1__Actinobacteria;D_2__Thermoleophilia;D_3__Gaiellales;D_4__uncultured | 0 | 0.013 | 0.008 | |
| D_0__Bacteria;D_1__Actinobacteria;D_2__Thermoleophilia;D_3__Solirubrobacterales;D_4__67-14 | 0.005 | 0.13 | 0 | |
| D_0__Bacteria;D_1__Actinobacteria;D_2__Thermoleophilia;D_3__Solirubrobacterales;D_4__Solirubrobacteraceae | 0.003 | 0 | 0.036 | |
| D_0__Bacteria;D_1__Actinobacteria;D_2__Thermoleophilia;D_3__Solirubrobacterales;__ | 0.005 | 0.008 | 0.093 | |
| D_0__Bacteria;D_1__Armatimonadetes;D_2__Armatimonadia;D_3__Armatimonadales;D_4__uncultured Armatimonadetes bacterium | 0 | 0.057 | 0.047 | |
| D_0__Bacteria;D_1__Armatimonadetes;D_2__Armatimonadia;D_3__Armatimonadales;D_4__uncultured bacterium | 0.013 | 0.017 | 0.011 | |
| D_0__Bacteria;D_1__Armatimonadetes;D_2__Fimbriimonadia;D_3__Fimbriimonadales;D_4__Fimbriimonadaceae | 0.021 | 0.03 | 0.005 | |
| D_0__Bacteria;D_1__Armatimonadetes;D_2__uncultured;D_3__uncultured bacterium;D_4__uncultured bacterium | 0.001 | 0.013 | 0.003 | |
| D_0__Bacteria;D_1__Bacteroidetes;D_2__Bacteroidia;D_3__Chitinophagales;D_4__Chitinophagaceae | 0.07 | 0.106 | 0.014 | |
| D_0__Bacteria;D_1__Bacteroidetes;D_2__Bacteroidia;D_3__Chitinophagales;D_4__Saprospiraceae | 0.028 | 0.111 | 0 | |
| D_0__Bacteria;D_1__Bacteroidetes;D_2__Bacteroidia;D_3__Chitinophagales;D_4__uncultured | 0 | 0.074 | 0.028 | |
| D_0__Bacteria;D_1__Bacteroidetes;D_2__Bacteroidia;D_3__Chitinophagales;__ | 0.027 | 0.021 | 0.066 | |
| D_0__Bacteria;D_1__Bacteroidetes;D_2__Bacteroidia;D_3__Cytophagales;D_4__Cyclobacteriaceae | 0.024 | 0.034 | 0 | |
| D_0__Bacteria;D_1__Bacteroidetes;D_2__Bacteroidia;D_3__Cytophagales;D_4__Cytophagaceae | 0.025 | 0.001 | 0.018 | |
| D_0__Bacteria;D_1__Bacteroidetes;D_2__Bacteroidia;D_3__Cytophagales;D_4__Hymenobacteraceae | 0.001 | 0.01 | 0 | |
| D_0__Bacteria;D_1__Bacteroidetes;D_2__Bacteroidia;D_3__Cytophagales;D_4__Microscillaceae | 0.01 | 0.003 | 0.01 | |
| D_0__Bacteria;D_1__Bacteroidetes;D_2__Bacteroidia;D_3__Cytophagales;D_4__Spirosomaceae | 0.037 | 0.018 | 0.003 | |
| D_0__Bacteria;D_1__Bacteroidetes;D_2__Bacteroidia;D_3__Cytophagales;__ | 0.004 | 0.029 | 0.08 | |
| D_0__Bacteria;D_1__Bacteroidetes;D_2__Bacteroidia;D_3__Flavobacteriales;D_4__Weeksellaceae | 0.005 | 0 | 0.039 | |
| D_0__Bacteria;D_1__Bacteroidetes;D_2__Bacteroidia;D_3__Sphingobacteriales;D_4__Sphingobacteriaceae | 0.006 | 0.043 | 0.021 | |
| D_0__Bacteria;D_1__Bacteroidetes;D_2__Bacteroidia;D_3__Sphingobacteriales;D_4__env.OPS 17 | 0.028 | 0.098 | 0.07 | |
| D_0__Bacteria;D_1__Bacteroidetes;D_2__Ignavibacteria;D_3__OPB56;D_4__uncultured bacterium | 0 | 0.076 | 0.027 | |
| D_0__Bacteria;D_1__Chloroflexi;D_2__Anaerolineae;D_3__Ardenticatenales;D_4__Ardenticatenaceae | 0.003 | 0.009 | 0 | |
| D_0__Bacteria;D_1__Chloroflexi;D_2__Anaerolineae;D_3__Caldilineales;D_4__Caldilineaceae | 0.008 | 0.033 | 0.082 | |
| D_0__Bacteria;D_1__Chloroflexi;D_2__Anaerolineae;D_3__SBR1031;D_4__A4b | 0.073 | 0.036 | 0.008 | |
| D_0__Bacteria;D_1__Chloroflexi;D_2__Chloroflexia;D_3__Chloroflexales;D_4__Chloroflexaceae | 0.053 | 0.027 | 0.028 | |
| D_0__Bacteria;D_1__Chloroflexi;D_2__Chloroflexia;D_3__Chloroflexales;D_4__Herpetosiphonaceae | 0 | 0.002 | 0.006 | |
| D_0__Bacteria;D_1__Chloroflexi;D_2__Chloroflexia;D_3__Chloroflexales;D_4__Roseiflexaceae | 0.004 | 0.031 | 0.003 | |
| D_0__Bacteria;D_1__Chloroflexi;D_2__Chloroflexia;D_3__Kallotenuales;D_4__AKIW781 | 0.003 | 0.008 | 0.016 | |
| D_0__Bacteria;D_1__Chloroflexi;D_2__Chloroflexia;D_3__Kallotenuales;__ | 0.008 | 0 | 0.002 | |
| D_0__Bacteria;D_1__Chloroflexi;D_2__Chloroflexia;D_3__Thermomicrobiales;D_4__AKYG1722 | 0.029 | 0.001 | 0.001 | |
| D_0__Bacteria;D_1__Chloroflexi;D_2__Chloroflexia;D_3__Thermomicrobiales;D_4__JG30-KF-CM45 | 0.001 | 0.085 | 0.01 | |
| D_0__Bacteria;D_1__Chloroflexi;D_2__JG30-KF-CM66;__;__ | 0.005 | 0.003 | 0.001 | |
| D_0__Bacteria;D_1__Chloroflexi;D_2__KD4-96;D_3__uncultured Chloroflexi bacterium;D_4__uncultured Chloroflexi bacterium | 0.044 | 0.01 | 0.008 | |
| D_0__Bacteria;D_1__Chloroflexi;D_2__KD4-96;__;__ | 0.007 | 0 | 0.032 | |
| D_0__Bacteria;D_1__Chloroflexi;D_2__Ktedonobacteria;D_3__C0119;D_4__uncultured Chloroflexi bacterium | 0 | 0 | 0.06 | |
| D_0__Bacteria;D_1__Chloroflexi;D_2__Ktedonobacteria;D_3__C0119;D_4__uncultured bacterium | 0.002 | 0.012 | 0 | |
| D_0__Bacteria;D_1__Chloroflexi;D_2__Ktedonobacteria;D_3__C0119;D_4__uncultured soil bacterium | 0.011 | 0.014 | 0.001 | |
| D_0__Bacteria;D_1__Chloroflexi;D_2__Ktedonobacteria;D_3__C0119;__ | 0.003 | 0.017 | 0.043 | |
| D_0__Bacteria;D_1__Chloroflexi;D_2__SHA-26;D_3__uncultured bacterium;__ | 0.044 | 0.123 | 0.007 | |
| D_0__Bacteria;D_1__Chloroflexi;D_2__TK10;D_3__metagenome;D_4__metagenome | 0.052 | 0 | 0.011 | |
| D_0__Bacteria;D_1__Chloroflexi;D_2__TK10;D_3__uncultured bacterium;D_4__uncultured bacterium | 0.001 | 0 | 0.045 | |
| D_0__Bacteria;D_1__Chloroflexi;D_2__TK10;__;__ | 0.009 | 0.035 | 0.01 | |
| D_0__Bacteria;D_1__Cyanobacteria;D_2__Oxyphotobacteria;D_3__Leptolyngbyales;D_4__Leptolyngbyaceae | 0.039 | 0.149 | 0.023 | |
| D_0__Bacteria;D_1__Cyanobacteria;D_2__Oxyphotobacteria;D_3__Nostocales;D_4__Chroococcidiopsaceae | 0.025 | 0.073 | 0.001 | |
| D_0__Bacteria;D_1__Cyanobacteria;D_2__Oxyphotobacteria;D_3__Nostocales;D_4__Coleofasciculaceae | 0.084 | **0.268** | 0.001 | |
| D_0__Bacteria;D_1__Cyanobacteria;D_2__Oxyphotobacteria;D_3__Nostocales;D_4__Cyanobacteriaceae | 0.001 | 0.016 | 0.026 | |
| D_0__Bacteria;D_1__Cyanobacteria;D_2__Oxyphotobacteria;D_3__Nostocales;D_4__Nostocaceae | 0.041 | 0.059 | 0.032 | |
| D_0__Bacteria;D_1__Cyanobacteria;D_2__Oxyphotobacteria;D_3__Nostocales;D_4__Phormidiaceae | 0.025 | 0.111 | 0.069 | |
| D_0__Bacteria;D_1__Cyanobacteria;D_2__Oxyphotobacteria;D_3__Nostocales;D_4__uncultured | 0.019 | 0.05 | 0.007 | |
| D_0__Bacteria;D_1__Cyanobacteria;D_2__Oxyphotobacteria;D_3__Nostocales;__ | 0.004 | 0.011 | 0.003 | |
| D_0__Bacteria;D_1__Cyanobacteria;D_2__Oxyphotobacteria;D_3__Oxyphotobacteria Incertae Sedis;D_4__Unknown Family | 0.043 | 0.077 | 0.012 | |
| D_0__Bacteria;D_1__Cyanobacteria;D_2__Oxyphotobacteria;D_3__Phormidesmiales;D_4__Nodosilineaceae | 0.008 | 0.01 | 0 | |
| D_0__Bacteria;D_1__Cyanobacteria;D_2__Oxyphotobacteria;D_3__RD011;D_4__uncultured cyanobacterium | 0.051 | 0.176 | 0.021 | |
| D_0__Bacteria;D_1__Cyanobacteria;D_2__Oxyphotobacteria;D_3__Thermosynechococcales;D_4__Thermosynechococcaceae | 0.038 | 0.025 | 0.012 | |
| D_0__Bacteria;D_1__Cyanobacteria;D_2__Oxyphotobacteria;__;__ | 0.008 | 0.025 | 0.002 | |
| D_0__Bacteria;D_1__Deinococcus-Thermus;D_2__Deinococci;D_3__Deinococcales;D_4__Trueperaceae | 0.002 | 0.007 | 0 | |
| D_0__Bacteria;D_1__FBP;D_2__uncultured actinobacterium;D_3__uncultured actinobacterium;D_4__uncultured actinobacterium | 0.002 | 0.024 | 0.026 | |
| D_0__Bacteria;D_1__FBP;D_2__uncultured bacterium;D_3__uncultured bacterium;D_4__uncultured bacterium | 0.005 | 0 | 0.005 | |
| D_0__Bacteria;D_1__FBP;D_2__uncultured endolithic bacterium;D_3__uncultured endolithic bacterium;D_4__uncultured endolithic bacterium | 0.036 | 0.005 | 0.088 | |
| D_0__Bacteria;D_1__FBP;D_2__uncultured organism;D_3__uncultured organism;D_4__uncultured organism | 0.007 | 0 | 0 | |
| D_0__Bacteria;D_1__FBP;D_2__uncultured soil bacterium;D_3__uncultured soil bacterium;D_4__uncultured soil bacterium | 0.001 | 0.003 | 0.154 | |
| D_0__Bacteria;D_1__FBP;__;__;__ | 0.006 | 0.028 | 0.006 | |
| D_0__Bacteria;D_1__Fibrobacteres;D_2__Fibrobacteria;D_3__Fibrobacterales;D_4__Fibrobacteraceae | 0.117 | 0.021 | 0 | |
| D_0__Bacteria;D_1__Firmicutes;D_2__Bacilli;D_3__Bacillales;D_4__Alicyclobacillaceae | 0.001 | 0.001 | 0.007 | |
| D_0__Bacteria;D_1__Firmicutes;D_2__Bacilli;D_3__Bacillales;D_4__Bacillaceae | 0.108 | 0.002 | 0.004 | |
| D_0__Bacteria;D_1__Firmicutes;D_2__Bacilli;D_3__Bacillales;D_4__Family XII | 0 | 0 | 0.075 | |
| D_0__Bacteria;D_1__Firmicutes;D_2__Bacilli;D_3__Bacillales;D_4__Paenibacillaceae | 0.017 | 0.054 | 0.001 | |
| D_0__Bacteria;D_1__Firmicutes;D_2__Bacilli;D_3__Bacillales;D_4__Planococcaceae | 0.016 | 0.005 | 0.03 | |
| D_0__Bacteria;D_1__Firmicutes;D_2__Bacilli;D_3__Bacillales;D_4__Thermoactinomycetaceae | | | | |
| D_0__Bacteria;D_1__Firmicutes;D_2__Bacilli;D_3__Bacillales;__ | | | | |
| D_0__Bacteria;D_1__Firmicutes;D_2__Clostridia;D_3__Clostridiales;D_4__Clostridiaceae 1 | 0.048 | 0.001 | 0.001 | |
| D_0__Bacteria;D_1__Gemmatimonadetes;D_2__Gemmatimonadetes;D_3__Gemmatimonadales;D_4__Gemmatimonadaceae | 0.01 | 0.015 | 0.006 | |
| D_0__Bacteria;D_1__Gemmatimonadetes;D_2__Longimicrobia;D_3__Longimicrobiales;D_4__Longimicrobiaceae | 0.002 | 0.001 | 0.003 | |
| D_0__Bacteria;D_1__Nitrospirae;D_2__Nitrospira;D_3__Nitrospirales;D_4__Nitrospiraceae | 0.002 | 0.028 | 0.001 | |
| D_0__Bacteria;D_1__Planctomycetes;D_2__Phycisphaerae;D_3__Tepidisphaerales;D_4__Tepidisphaeraceae | 0.05 | 0.028 | 0.001 | |
| D_0__Bacteria;D_1__Planctomycetes;D_2__Phycisphaerae;D_3__Tepidisphaerales;D_4__WD2101 soil group | 0.032 | 0.009 | 0.004 | |
| D_0__Bacteria;D_1__Planctomycetes;D_2__Planctomycetacia;D_3__Gemmatales;D_4__Gemmataceae | 0.001 | 0.021 | 0 | |
| D_0__Bacteria;D_1__Planctomycetes;D_2__Planctomycetacia;D_3__Isosphaerales;D_4__Isosphaeraceae | 0.016 | 0.004 | 0 | |
| D_0__Bacteria;D_1__Planctomycetes;D_2__Planctomycetacia;D_3__Pirellulales;D_4__Pirellulaceae | 0.016 | 0.014 | 0.048 | |
| D_0__Bacteria;D_1__Planctomycetes;D_2__vadinHA49;__;__ | 0.025 | 0.074 | 0.011 | |
| D_0__Bacteria;D_1__Proteobacteria;D_2__Alphaproteobacteria;D_3__Acetobacterales;D_4__Acetobacteraceae | 0.007 | 0 | 0.007 | |
| D_0__Bacteria;D_1__Proteobacteria;D_2__Alphaproteobacteria;D_3__Azospirillales;D_4__Azospirillaceae | 0.012 | 0.02 | 0.075 | |
| D_0__Bacteria;D_1__Proteobacteria;D_2__Alphaproteobacteria;D_3__Caulobacterales;D_4__Caulobacteraceae | 0.003 | 0.002 | 0.008 | |
| D_0__Bacteria;D_1__Proteobacteria;D_2__Alphaproteobacteria;D_3__Elsterales;D_4__uncultured | 0.024 | 0.007 | 0 | |
| D_0__Bacteria;D_1__Proteobacteria;D_2__Alphaproteobacteria;D_3__Puniceispirillales;D_4__Puniceispirillales Incertae Sedis | 0.008 | 0.026 | 0.026 | |
| D_0__Bacteria;D_1__Proteobacteria;D_2__Alphaproteobacteria;D_3__Rhizobiales;D_4__Beijerinckiaceae | 0 | 0.015 | 0.001 | |
| D_0__Bacteria;D_1__Proteobacteria;D_2__Alphaproteobacteria;D_3__Rhizobiales;D_4__D05-2 | 0.01 | 0.161 | 0.019 | |
| D_0__Bacteria;D_1__Proteobacteria;D_2__Alphaproteobacteria;D_3__Rhizobiales;D_4__Devosiaceae | 0.003 | 0.053 | 0.012 | |
| D_0__Bacteria;D_1__Proteobacteria;D_2__Alphaproteobacteria;D_3__Rhizobiales;D_4__Labraceae | 0 | 0.083 | 0.001 | |
| D_0__Bacteria;D_1__Proteobacteria;D_2__Alphaproteobacteria;D_3__Rhizobiales;D_4__Methylopilaceae | 0.035 | 0.02 | 0.001 | |
| D_0__Bacteria;D_1__Proteobacteria;D_2__Alphaproteobacteria;D_3__Rhizobiales;D_4__Rhizobiaceae | 0.005 | 0.041 | 0.026 | |
| D_0__Bacteria;D_1__Proteobacteria;D_2__Alphaproteobacteria;D_3__Rhizobiales;D_4__Rhodomicrobiaceae | 0.005 | 0.075 | 0.003 | |
| D_0__Bacteria;D_1__Proteobacteria;D_2__Alphaproteobacteria;D_3__Rhizobiales;D_4__Xanthobacteraceae | 0.005 | 0.07 | 0.024 | |
| D_0__Bacteria;D_1__Proteobacteria;D_2__Alphaproteobacteria;D_3__Rhizobiales;D_4__uncultured | 0 | 0.1 | 0.108 | |
| D_0__Bacteria;D_1__Proteobacteria;D_2__Alphaproteobacteria;D_3__Rhizobiales;__ | 0.062 | 0.022 | 0.048 | |
| D_0__Bacteria;D_1__Proteobacteria;D_2__Alphaproteobacteria;D_3__Rhodobacterales;D_4__Rhodobacteraceae | 0.003 | 0.03 | 0.002 | |
| D_0__Bacteria;D_1__Proteobacteria;D_2__Alphaproteobacteria;D_3__Sphingomonadales;D_4__Sphingomonadaceae | 0.006 | 0.043 | 0.02 | |
| D_0__Bacteria;D_1__Proteobacteria;D_2__Alphaproteobacteria;D_3__Tistrellales;D_4__Geminicoccaceae | 0.008 | 0.011 | 0.03 | |
| D_0__Bacteria;D_1__Proteobacteria;D_2__Alphaproteobacteria;D_3__uncultured;__ | 0.029 | 0 | 0.004 | |
| D_0__Bacteria;D_1__Proteobacteria;D_2__Alphaproteobacteria;__;__ | 0.015 | 0.012 | 0.003 | |
| D_0__Bacteria;D_1__Proteobacteria;D_2__Deltaproteobacteria;D_3__Bdellovibrionales;D_4__Bdellovibrionaceae | 0.002 | 0 | 0.014 | |
| D_0__Bacteria;D_1__Proteobacteria;D_2__Deltaproteobacteria;D_3__Myxococcales;D_4__Archangiaceae | 0.015 | 0.001 | 0 | |
| D_0__Bacteria;D_1__Proteobacteria;D_2__Deltaproteobacteria;D_3__Myxococcales;D_4__BIrii41 | 0.051 | 0.18 | 0.002 | |
| D_0__Bacteria;D_1__Proteobacteria;D_2__Deltaproteobacteria;D_3__Myxococcales;D_4__Haliangiaceae | 0.029 | 0.01 | 0 | |
| D_0__Bacteria;D_1__Proteobacteria;D_2__Deltaproteobacteria;D_3__Myxococcales;D_4__Myxococcaceae | 0.006 | 0 | 0.003 | |
| D_0__Bacteria;D_1__Proteobacteria;D_2__Deltaproteobacteria;D_3__Myxococcales;D_4__Nannocystaceae | 0.028 | 0.037 | 0.002 | |
| D_0__Bacteria;D_1__Proteobacteria;D_2__Deltaproteobacteria;D_3__Myxococcales;D_4__P3OB-42 | 0.022 | 0.011 | 0.007 | |
| D_0__Bacteria;D_1__Proteobacteria;D_2__Deltaproteobacteria;D_3__Myxococcales;D_4__Phaselicystidaceae | 0.011 | 0.002 | 0.002 | |
| D_0__Bacteria;D_1__Proteobacteria;D_2__Deltaproteobacteria;D_3__Myxococcales;D_4__Polyangiaceae | 0.031 | 0 | 0.014 | |
| D_0__Bacteria;D_1__Proteobacteria;D_2__Deltaproteobacteria;D_3__Myxococcales;D_4__Sandaracinaceae | 0.002 | **0.222** | 0.011 | |
| D_0__Bacteria;D_1__Proteobacteria;D_2__Deltaproteobacteria;D_3__Myxococcales;D_4__bacteriap25 | 0.014 | 0.026 | 0.006 | |
| D_0__Bacteria;D_1__Proteobacteria;D_2__Deltaproteobacteria;D_3__Myxococcales;D_4__uncultured bacterium | 0 | 0.005 | 0.004 | |
| D_0__Bacteria;D_1__Proteobacteria;D_2__Deltaproteobacteria;D_3__Myxococcales;__ | 0.072 | 0 | 0.019 | |
| D_0__Bacteria;D_1__Proteobacteria;D_2__Deltaproteobacteria;D_3__Oligoflexales;D_4__Oligoflexaceae | 0.019 | 0.031 | 0 | |
| D_0__Bacteria;D_1__Proteobacteria;D_2__Deltaproteobacteria;D_3__SAR324 clade(Marine group B);D_4__uncultured soil bacterium | 0.043 | 0 | 0 | |
| D_0__Bacteria;D_1__Proteobacteria;D_2__Gammaproteobacteria;D_3__Betaproteobacteriales;D_4__Burkholderiaceae | 0.006 | 0.037 | 0.019 | |
| D_0__Bacteria;D_1__Proteobacteria;D_2__Gammaproteobacteria;D_3__Betaproteobacteriales;D_4__TRA3-20 | 0.019 | **0.251** | 0.024 | |
| D_0__Bacteria;D_1__Proteobacteria;D_2__Gammaproteobacteria;D_3__Cellvibrionales;D_4__Cellvibrionaceae | 0 | 0.005 | 0.001 | |
| D_0__Bacteria;D_1__Proteobacteria;D_2__Gammaproteobacteria;D_3__Enterobacteriales;D_4__Enterobacteriaceae | 0.003 | 0.003 | 0.001 | |
| D_0__Bacteria;D_1__Proteobacteria;D_2__Gammaproteobacteria;D_3__Nitrosococcales;D_4__Nitrosococcaceae | 0.001 | 0.005 | 0.027 | |
| D_0__Bacteria;D_1__Proteobacteria;D_2__Gammaproteobacteria;D_3__Oceanospirillales;D_4__Pseudohongiellaceae | 0.026 | **0.239** | 0.001 | |
| D_0__Bacteria;D_1__Proteobacteria;D_2__Gammaproteobacteria;D_3__Pseudomonadales;D_4__Moraxellaceae | 0.021 | 0.005 | 0.031 | |
| D_0__Bacteria;D_1__Proteobacteria;D_2__Gammaproteobacteria;D_3__Pseudomonadales;D_4__Pseudomonadaceae | 0 | 0.008 | 0 | |
| D_0__Bacteria;D_1__Proteobacteria;D_2__Gammaproteobacteria;D_3__Steroidobacterales;D_4__Steroidobacteraceae | 0.005 | 0.002 | 0.002 | |
| D_0__Bacteria;D_1__Proteobacteria;D_2__Gammaproteobacteria;D_3__Xanthomonadales;D_4__Rhodanobacteraceae | 0.029 | 0.003 | 0 | |
| D_0__Bacteria;D_1__Proteobacteria;__;__;__ | 0.007 | 0.009 | 0.098 | |
| D_0__Bacteria;D_1__Verrucomicrobia;D_2__Verrucomicrobiae;D_3__Chthoniobacterales;D_4__Chthoniobacteraceae | 0.004 | 0.044 | 0.003 | |
| D_0__Bacteria;D_1__Verrucomicrobia;D_2__Verrucomicrobiae;D_3__Opitutales;D_4__Opitutaceae | 0.07 | 0.183 | 0 | |
| D_0__Bacteria;D_1__Verrucomicrobia;D_2__Verrucomicrobiae;D_3__Pedosphaerales;D_4__Pedosphaeraceae | 0.038 | 0.221 | 0.026 | |
| D_0__Bacteria;D_1__Verrucomicrobia;D_2__Verrucomicrobiae;D_3__Verrucomicrobiales;D_4__Akkermansiaceae | 0.003 | 0.004 | 0.002 | |
| D_0__Bacteria;D_1__Verrucomicrobia;D_2__Verrucomicrobiae;D_3__Verrucomicrobiales;D_4__Verrucomicrobiaceae | 0.03 | **0.196** | 0.001 | |
| D_0__Bacteria;__;__;__;__ | 0.009 | 0.007 | 0.036 | |
|  |  |  |  | |
| **b.**  **Biocrust cover categories – correlations with Bacterial NMDS (Fig. 1b)** | **Axis 1 (x)** | **Axis 2 (y)** | **Axis 3** | |
|  | **r-sq** | **r-sq** | **r-sq** | |
| Dark Cyanobacteria | 0.035 | 0.178 | 0.22 | |
| Light Cyanobacteria | 0 | 0.34 | 0.012 | |
| *Syntrichia ruralis* | 0.08 | 0.115 | 0.046 | |
| *Syntrichia caninervis* | 0.032 | 0.042 | **0.281** | |
| *Bryum argenteum* | 0.013 | 0.106 | 0.006 | |
| *Gemmabryum caespiticium* | 0.044 | 0.101 | 0.011 | |
| *Gemmabryum kunzei* | 0.027 | 0.019 | 0 | |
| *Encalypta vulgaris* | 0.003 | 0.016 | 0.01 | |
| *Pterygoneurum sp.* | 0.004 | 0.006 | 0.003 | |
| *Enchylium tenax* | 0.172 | 0.003 | 0.018 | |
| *Enchylium coccophorum* | 0.025 | 0 | 0.001 | |
| *Candelariella sp.* | 0.008 | 0.04 | 0.058 | |
| *Gyalolechia sp.* | 0 | 0.08 | 0.125 | |
| *Diploschistes muscorum* | 0.036 | 0.01 | 0.015 | |
| *Placidium squamulosum* | 0.066 | 0 | 0.007 | |
| *Psora sp.* | 0.003 | 0.007 | 0.017 | |
| *Clavascidium lacinulatum* | 0.063 | 0 | 0.001 | |
| *Aspicilia sp.* | 0.002 | 0.004 | 0.002 | |
| *Endocarpon sp.* | 0.032 | 0.009 | 0.008 | |
| *Toninia sp.* | 0.003 | 0.001 | 0.009 | |
| *Peltula sp.* | 0.005 | 0.001 | 0.038 | |
| *Acarospora sp.* | 0.004 | 0.014 | 0.004 | |
|  |  |  | |  |
| **c. Biocrust visible cover community - correlations with Cyanobacterial NMDS (Fig. 1c)** | **Axis 1 (x)** | **Axis 2** | | **Axis 3 (y)** |
|  | **r-sq** | **r-sq** | | **r-sq** |
| Dark Cyanobacteria | 0.021 | 0 | | **0.219** |
| Light Cyanobacteria | 0.011 | 0.006 | | **0.277** |
| *Syntrichia ruralis* | 0.04 | 0 | | 0.09 |
| *Syntrichia caninervis* | 0.033 | 0.006 | | 0.07 |
| *Bryum argenteum* | 0.067 | 0 | | 0.083 |
| *Gemmabryum caespiticium* | 0.046 | 0.004 | | 0.116 |
| *Bryum kunzei* | 0.015 | 0.008 | | 0.02 |
| *Encalypta vulgaris* | 0.001 | 0 | | 0.038 |
| *Pterygoneurum sp.* | 0.003 | 0.016 | | 0.004 |
| *Enchylium tenax* | 0.11 | 0.016 | | 0.009 |
| *Enchylium coccophorum* | 0.03 | 0.034 | | 0.027 |
| *Candelariella sp.* | 0.004 | 0.003 | | 0.037 |
| *Gyalolechia sp.* | 0.004 | 0.001 | | 0.114 |
| *Diploschistes muscorum* | 0.02 | 0.006 | | 0.021 |
| *Placidium squamulosum* | 0.071 | 0.006 | | 0 |
| *Psora sp.* | 0.001 | 0.025 | | 0.009 |
| *Clavascidium lacinulatum* | 0.048 | 0.011 | | 0.005 |
| *Aspicilia sp.* | 0 | 0 | | 0 |
| *Endocarpon sp.* | 0.032 | 0.02 | | 0.006 |
| *Toninia sp.* | 0.002 | 0.003 | | 0.001 |
| *Peltula sp.* | 0.003 | 0.032 | | 0.022 |
| *Acarospora sp.* | 0.001 | 0.014 | | 0.001 |
|  |  |  | |  |
|  |  |  | |  |
|  |  |  | |  |
|  |  |  | |  |
|  |  |  | |  |
|  |  |  | |  |
|  |  |  | |  |
|  |  |  | |  |

Figures

Figure S1. Images of experimental garden set up. Top row shows the low elevation (a), mid elevation (b) and high elevation (c) gardens. In the middle row, we see examples of biocrust experimental units (d-f) and the installation process (g). The bottom row are close ups showing the variety of community assemblies of individual units (h-k).


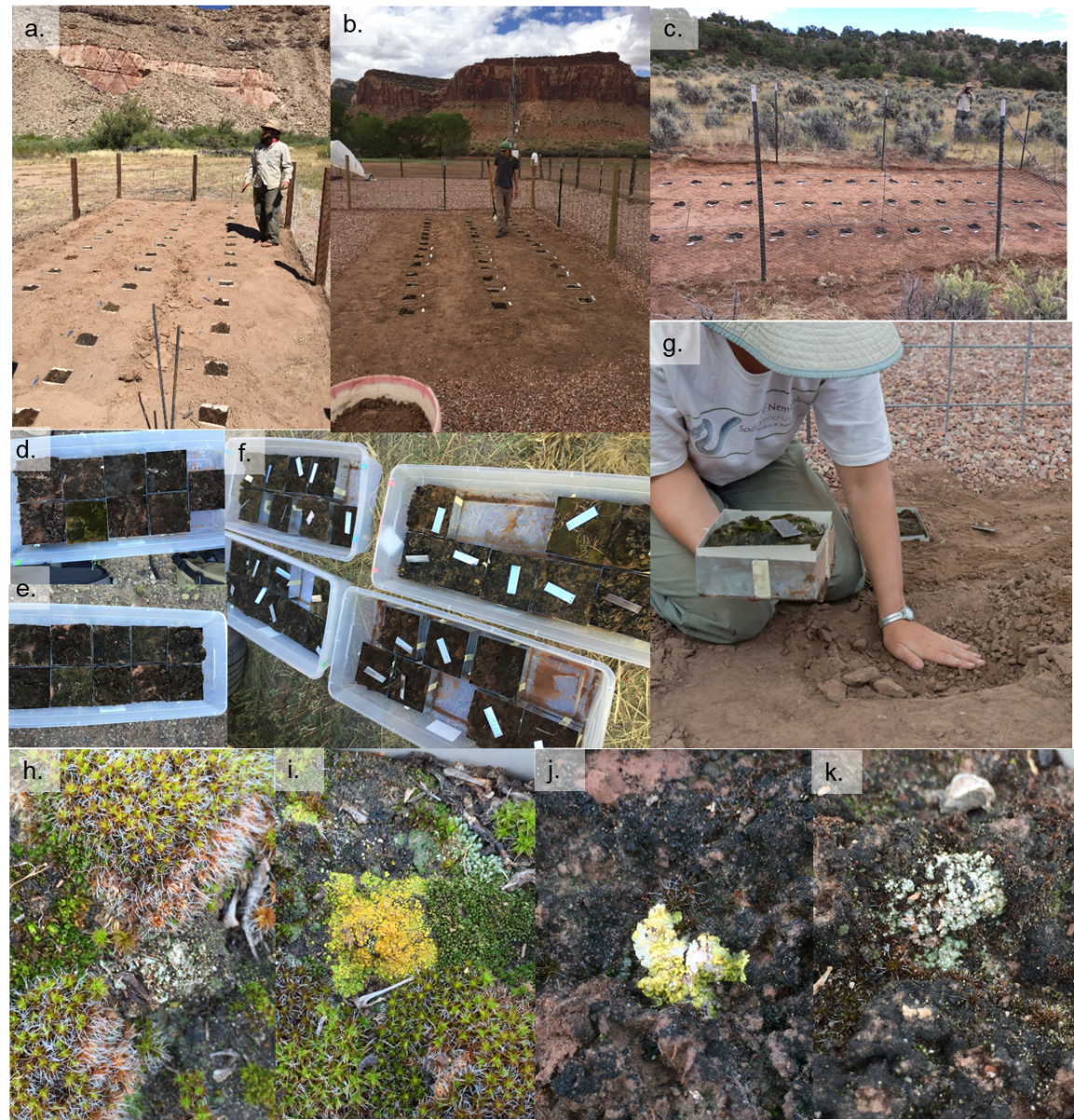


Figure S2. Rarefaction curve by sample and by treatment. Sequences were rarified at 14,595 sequences to preserve maximum diversity and number of samples.


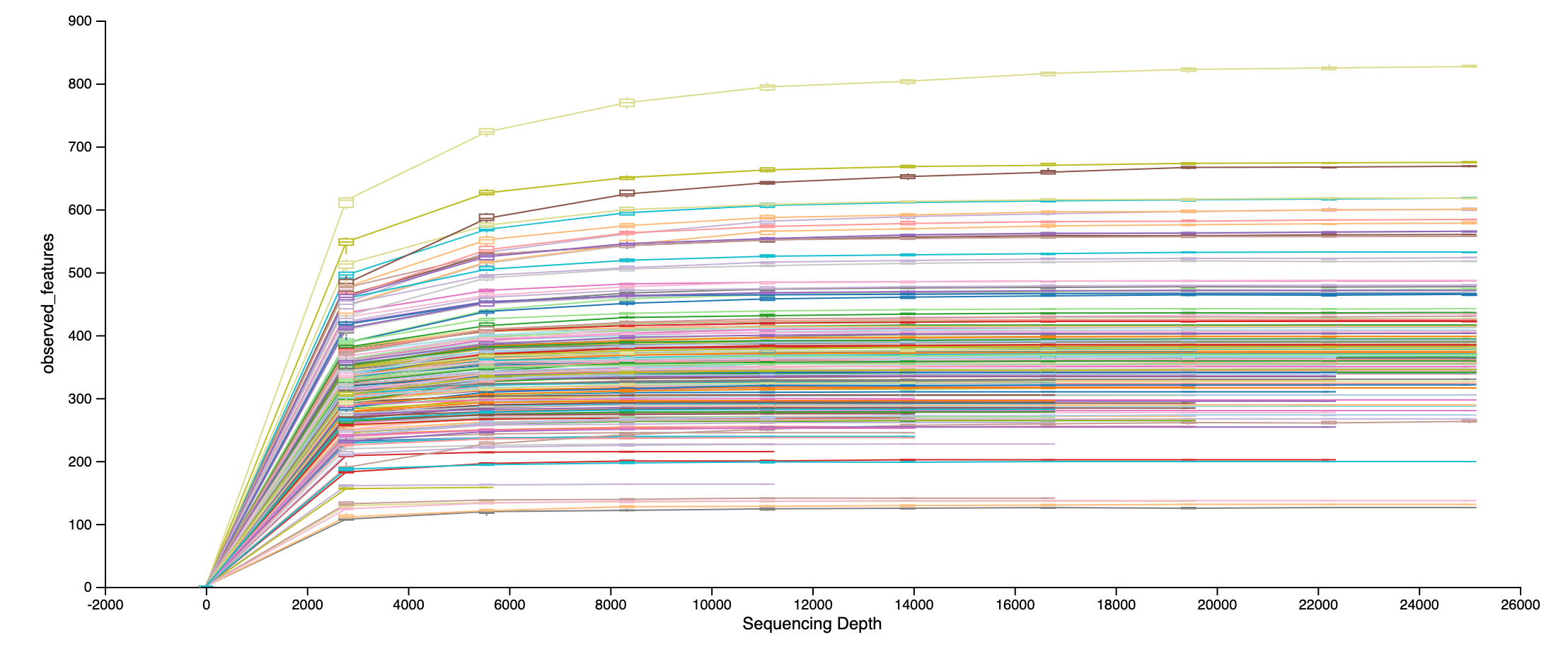


Figure S3. Biocrust visible cover by morphospecies communities at start (left) and end points (right). Species are grouped by mosses (green), lichens (brown) and cyanobacteria (purple).


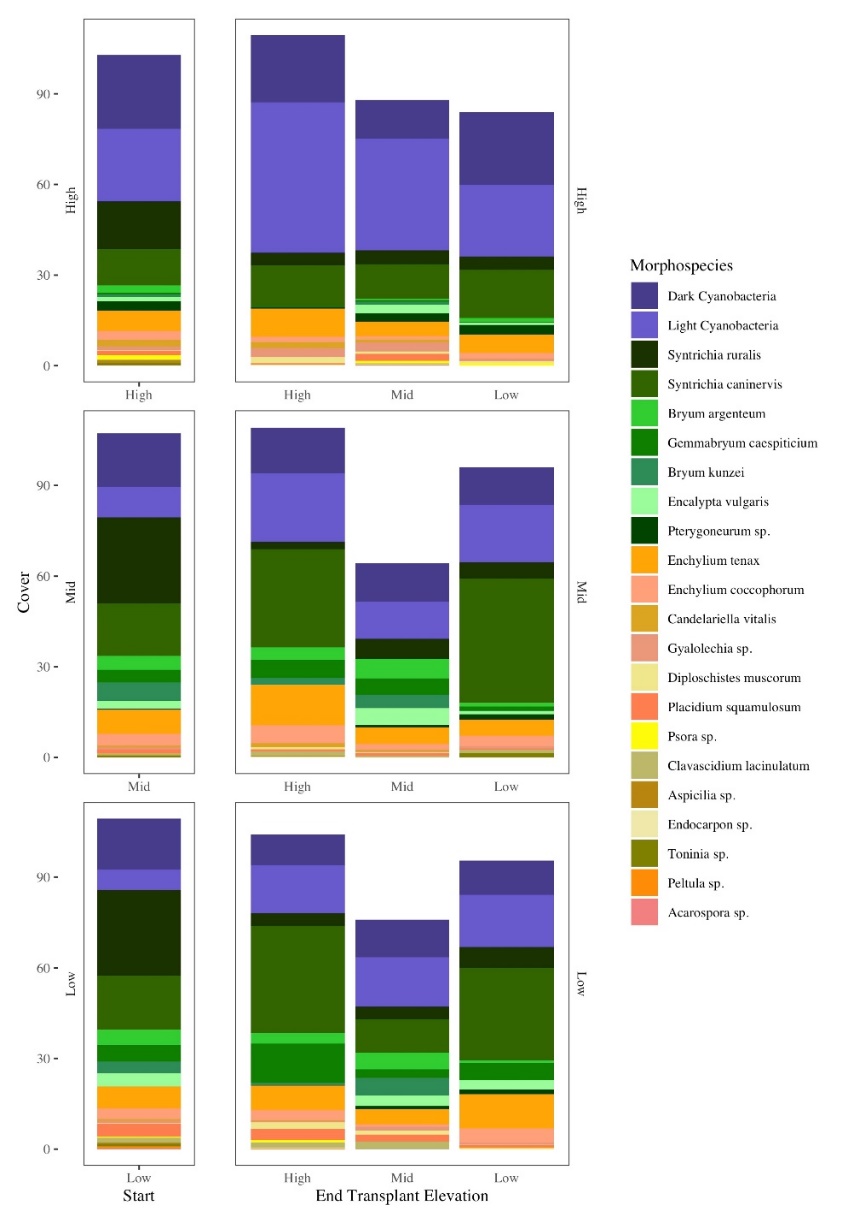


Figure S4 – Total soil bacterial start (left) and end communities (right) by phylum.


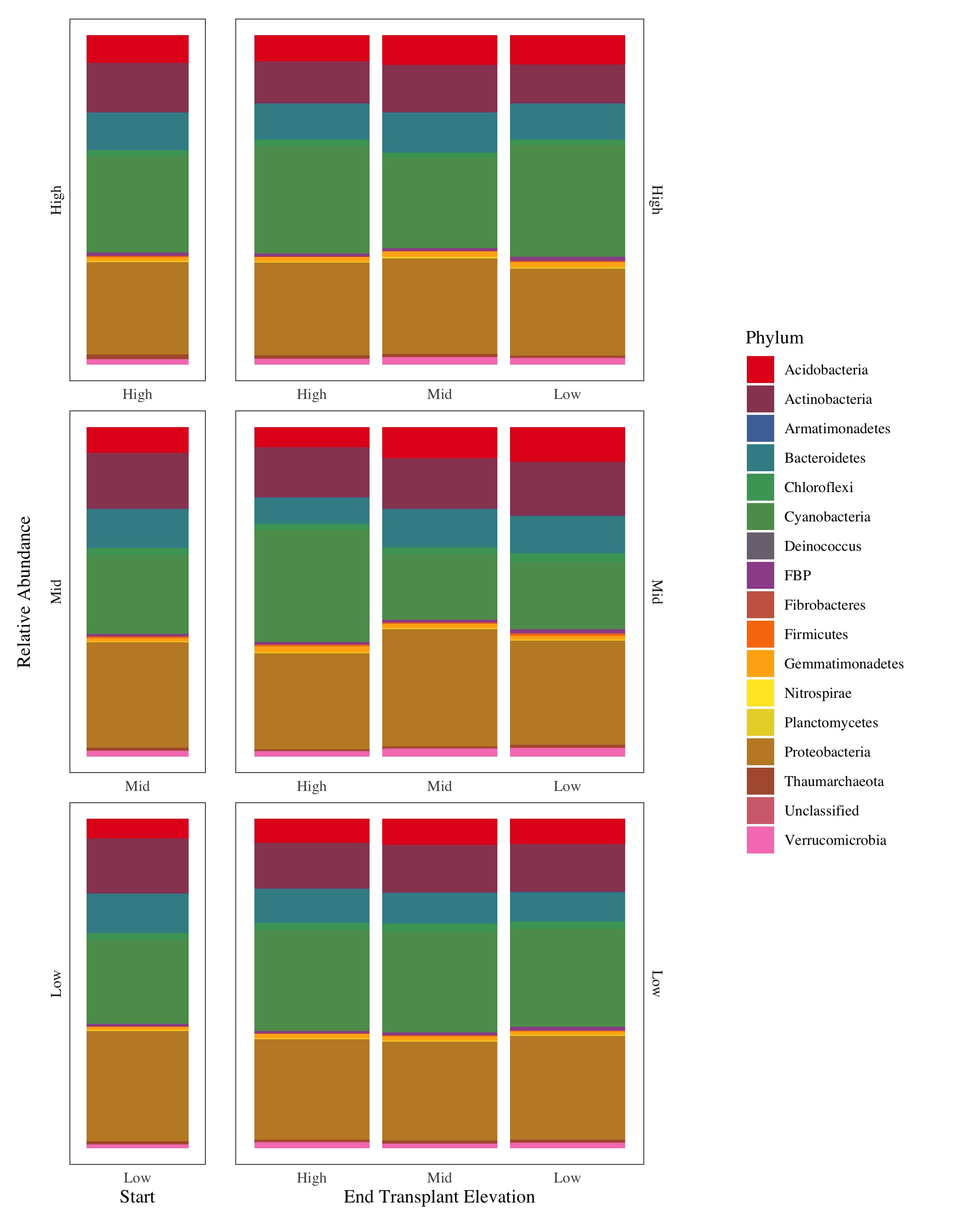

Supplement: Supplementary file 1 [file Data_Sheet_1.docx]
